# MXene‐Based Materials for Electrochemical Sodium‐Ion Storage

**DOI:** 10.1002/advs.202003185

**Published:** 2021-03-15

**Authors:** Pin Ma, Daliang Fang, Yilin Liu, Yang Shang, Yumeng Shi, Hui Ying Yang

**Affiliations:** ^1^ International Collaborative Laboratory of 2D Materials for Optoelectronics Science and Technology of Ministry of Education Institute of Microscale Optoelectronics Shenzhen University Shenzhen 518060 China; ^2^ Pillar of Engineering Product Development Singapore University of Technology and Design 8 Somapah Road Singapore 487372 Singapore; ^3^ Engineering Technology Research Center for 2D Material Information Function Devices and Systems of Guangdong Province College of Optoelectronic Engineering Shenzhen University Shenzhen 518060 China

**Keywords:** electrochemical storage, electrode materials, MXene, sodium‐ion batteries, sodium‐ion capacitors, sodium–sulfur batteries

## Abstract

Advanced architecture and rational design of electrode materials for electrochemical sodium‐ion storage are well developed by researchers worldwide. MXene‐based materials are considered as one of the most potential electrode materials for sodium‐ion‐based devices, such as sodium‐ion batteries (SIBs), sodium–sulfur batteries (SSBs), and sodium‐ion capacitors (SICs), because of the excellent physicochemical characteristics of MXenes. Here, in this review, the recent research work and progress, both theoretical and experimental, on MXene‐based materials including pure MXenes and MXene‐based composites in application of SIBs, SSBs, and SICs are comprehensively summarized. The sodium storage mechanisms and the effective methods to enhance the electrochemical performance are also discussed. Finally, the current critical challenges and future research directions on the development of these MXene‐based materials for electrochemical sodium‐ion storage are presented.

## Graphical abstract

This review provides an in‐depth summary of the application of MXene‐based materials in the sodium‐ion storage, including the detailed sodium‐ion storage performances and mechanisms. Moreover, the relation between the structures and electrochemical performances and the corresponding effective strategies for further optimization are also discussed. In addition, the perspectives on dealing with current challenges are proposed.

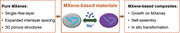

## 1 Introduction

Sodium‐ion storage is the strong alternative to lithium‐ion storage for large‐scale renewable energy storage systems due to the similar physical/chemical properties, higher elemental abundance, and lower supply cost of sodium to lithium. Unfortunately, compared with lithium, sodium has larger ion radius (0.102 nm), higher standard reduction potential (−2.71 V vs standard hydrogen electrode (SHE)), and lower electronegativity (0.93), leading to the sluggish sorption or/and insertion kinetics and large volume expansion.^[^ ^1^ ^]^ Therefore, the sodium‐ion‐based devices, such as sodium‐ion batteries (SIBs), sodium–sulfur batteries (SSBs), and sodium‐ion capacitors (SICs), always suffer from the low reversible capacity and poor cycling stability.^[^ ^2^ ^]^ Thus, new chemical structure and architecture of sodium accommodable materials should be developed to improve the efficiency of sodium storage.

Recently, a new large group of 2D transition metal carbides, carbonitrides, and nitrides labeled as MXenes has attracted tremendous attention.^[^ ^3^ ^]^ The MXenes family shares a general composition of M*_n_*_+1_X*_n_*T*_x_*, where M represents a transition metal like Ti, V, Mn, Mo, Nb, Cr, Sc, etc., X is C and/or N, and T*_x_* stands for terminal surface groups ‐O, ‐F, and/or ‐OH, which is typically prepared by selectively etching of A layers, such as Al, Si, Ga, from the corresponding M*_n_*_+1_AX*_n_* phases. Owing to their outstanding metallic conductivity, tunable surface chemistry, and 2D layered structure, MXenes have been considered as the promising candidates for supercapacitors,^[^ ^4^ ^]^ rechargeable batteries,^[^ ^5^, ^6^ ^]^ catalysts,^[^ ^7^ ^]^ oxygen reduction and evolution,^[^ ^8^ ^]^ water purification,^[^ ^9^ ^]^ electromagnetic interference shielding,^[^ ^10^ ^]^ pressure sensor,^[^ ^11^ ^]^ and field‐effect transistors^[^ ^12^ ^]^ applications.

In fact, many research have been conducted on MXenes as electrode materials for sodium‐ion‐based devices.^[^ ^13^, ^14^, ^15^, ^16^, ^17^, ^18^ ^]^ Based on the density functional theory (DFT) calculations,^[^ ^19^, ^20^ ^]^ the surface termination groups have a great effect on the properties and performance of MXenes. For example, sodium ions can be well adsorbed on monolayer bare MXenes or O‐terminated MXenes due to the good negative adsorption energies but cannot well absorbed on the monolayer F‐terminated and OH‐terminated MXenes. Simultaneously, the monolayer bare MXenes or O‐terminated MXenes also exhibit low diffusion barrier and open‐circuit voltage (OCV) for sodium ions, suggesting that they are expected to be the promising anode materials with high capacities and good rate capabilities. However, experimentally, MXenes have the tendency to aggregate or stack, which impedes the charge transport through the electrodes, resulting in limited capacity values. To address these issues, many strategies including single‐/few‐layer MXenes, expanded interlayer spacing, 3D porous structures have been proposed to accelerate the electrochemical kinetics and enhance the capability.

Additionally, compared with the existing anode materials for SIBs, pure MXene electrodes do not perform the satisfying reversible capacity, limiting their further application in energy storage fields.^[^ ^2^, ^21^ ^]^ Therefore, many researchers have focused on designing the MXene‐based composite materials. For one thing, MXenes can offer the intertwined conductive network and then significantly increase the electronic conductivity. For another, secondary materials are expected to prevent the aggregation of individual nanosheets. What is more, the unique structures and synergistic effects are beneficial for the electrochemical performance.

**Figure 1:**
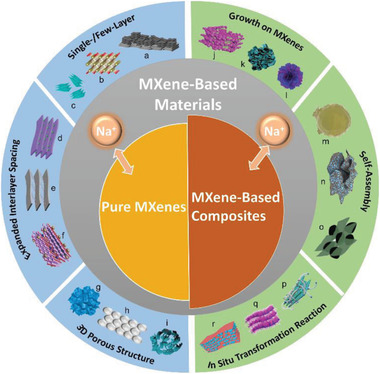
MXene‐based materials for sodium‐ion storage. (a) Reproduced with permission.[83] Copyright 2018, American Chemical Society. (b) Reproduced with permission.[15] Copyright 2017, American Chemical Society. (c) Reproduced with permission.[81] Copyright 2017, American Chemical Society. (d) Reproduced with permission.[84] Copyright 2018, Royal Society of Chemistry. (e) Reproduced with permission.[88] Copyright 2018, WILEY‐VCH GmbH. (f) Reproduced with permission.[85] Copyright 2019, Elsevier B.V. (g) Reproduced with permission.[14] Copyright 2020, American Chemical Society. (h) Reproduced with permission.[94] Copyright 2017, WILEY‐VCH GmbH. (i) Reproduced with permission.[90] Copyright 2018, Royal Society of Chemistry. (j) Reproduced with permission.[105] Copyright 2020, Royal Society of Chemistry. (k) Reproduced with permission.[102] Copyright 2019, Royal Society of Chemistry. (l) Reproduced with permission.[110] Copyright 2019, Royal Society of Chemistry. (m) Reproduced with permission.[118] Copyright 2018, Elsevier B.V. (n) Reproduced with permission.[114] Copyright 2018, American Chemical Society. (o) Reproduced with permission.[121] Copyright 2019, WILEY‐VCH GmbH. (p) Reproduced with permission.[132] Copyright 2020, Royal Society of Chemistry. (q) Reproduced with permission.[130] Copyright 2018, Elsevier Ltd. (r) Reproduced with permission.[129] Copyright 2018, Royal Society of Chemistry.

In this review, the recent research work and progress carried out on the MXene‐based materials for sodium‐ion storage are systematically and comprehensively summarized. We put the emphasis on their synthesis conditions, structures, ion intercalation chemistries, and detailed sodium‐ion storage performances based on the experimental and theoretical investigations. Particularly, the application of pure MXenes and MXene‐based composite materials in the electrodes for SIBs, SSBs, and SICs are both introduced, and the corresponding effective methods to optimize their performance are highlighted and discussed in depth (**Figure** **1**). In addition, sodium‐ion storage mechanisms along with the relation between the structures and electrochemical performance are also intensively revealed. Finally, conclusions and our perspectives on current challenges and future directions of MXene‐based materials for electrochemical sodium‐ion storage are proposed.

## 2 Synthetic Strategies for MXenes

### 2.1 F‐Containing Etching Method

**Figure 2:**
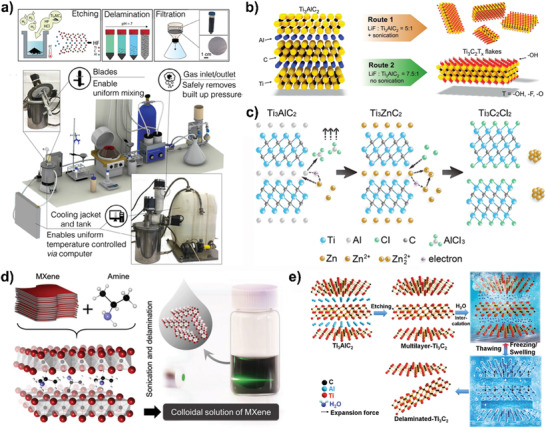
a) Schematic of MXene synthesis, images of 1 L MXene reactor and 3D model of synthesis setup. Reproduced with permission.^[^ ^36^ ^]^ Copyright 2020, WILEY‐VCH GmbH. b) Synthesis of Ti_3_C_2_T*_x_* flakes produced by different routes. Reproduced with permission.^[^ ^37^ ^]^ Copyright 2016, WILEY‐VCH GmbH. c) Synthesis of Ti_3_C_2_Cl_2_ by reaction with Lewis acidic molten salts. Reproduced with permission.^[^ ^40^ ^]^ Copyright 2019, American Chemical Society. d) Schematic of Nb_2_CT*_x_* delamination process via isopropylamine interaction. Reproduced with permission.^[^ ^26^ ^]^ Copyright 2015, WILEY‐VCH GmbH. e) The schematic diagram of the preparation of the delaminated‐Ti_3_C_2_ via the FAT method. Reproduced with permission.^[^ ^44^ ^]^ Copyright 2020, WILEY‐VCH GmbH.

It has been demonstrated that the synthesis conditions used to prepare MXenes are strongly dependent on the precursor, etchant, etching time, and temperature, directly influencing their physical/chemical characteristics and performance in applications.^[^ ^22^ ^]^ Since their first discovery in 2011,^[^ ^2^, ^21^ ^]^ over 20 species of MXenes have been created such as Ti_3_C_2_,^[^ ^23^, ^24^ ^]^ Ti_2_C,^[^ ^25^ ^]^ Nb_2_C,^[^ ^26^ ^]^ Nb_4_C_3_,^[^ ^27^ ^]^ V_2_C,^[^ ^28^, ^29^ ^]^ Mo_2_C,^[^ ^23^, ^30^ ^]^ Zr_3_C_2_,^[^ ^31^ ^]^ Hf_3_C_2_,^[^ ^15^ ^]^ Ta_4_C_3_,^[^ ^25^ ^]^ Mo_2_TiC_2_,^[^ ^32^ ^]^ Ti_4_N_3_,^[^ ^33^ ^]^ V_2_N.^[^ ^34^ ^]^ They are currently mainly synthesized by selectively etching of A layers in the corresponding MAX phases by various etchants, including aqueous hydrofluoric acid (HF),^[^ ^23^ ^]^ ammonium bifluoride (NH_4_HF_2_),^[^ ^35^ ^]^ LiF‐HCl (in situ forming HF),^[^ ^24^ ^]^ etc., resulting in the mixture of O‐, OH‐, and F‐terminated functional groups. For instance, the exfoliated 2D Ti_3_C_2_T*_x_* with accordion‐like morphology could be obtained by treating the Ti_3_AlC_2_ powders in HF for 2 h.^[^ ^23^ ^]^ The large‐volume synthesis of Ti_3_C_2_T*_x_* using this method (**Figure** **2**a) has no effect on the morphology and properties of materials, suggesting that scaling the production of MXenes to future commercialization is feasible.^[^ ^36^ ^]^ Exploiting the mixture of HCl and LiF could in situ form HF and produce clay‐like Ti_3_C_2_T*_x_*.^[^ ^24^ ^]^ Through increasing the molar ratio of MAX:LiF to 1:7.5 to provide excess of lithium for intercalation and replacing sonication with manual shaking to delaminate (Figure 2b), the synthesized Ti_3_C_2_T*_x_* flakes showed higher quality with free holes and larger size with well‐defined edges.^[^ ^37^ ^]^

In addition, MXenes can also be created from non‐Al‐based MAX phases by hazardous F‐containing solutions. For example, with assistance of oxidant, Ti_3_C_2_T*_x_* could be synthesized from Si‐based precursors of Ti_3_SiC_2_.^[^ ^38^ ^]^ A new strategy called high‐energy ultrasonic cell crushing extraction was also adopted to synthesize Ti_3_C_2_T*_x_* from Ti_3_SiC_2_, accelerating the preparation process and improving the extraction efficiency.^[^ ^39^ ^]^ Selectively etching of Ga from nanolaminated Mo_2_Ga_2_C using HF or LiF‐HCl brought about the 2D Mo_2_CT*_x_* flakes.^[^ ^30^ ^]^

### 2.2 F‐Free Etching Method

Compared with the hazardous F‐containing solutions, exploring safe and environmental friendly synthesis routes is highly desired for synthesizing MXenes, which can also give different functional groups like ‐O, ‐OH, and/or ‐Cl. A 2D hydroxyl‐terminated scandium carbide (ScC*_x_*OH) could be obtained by selectively etching the ScAl_3_C_3_ precursor using the organic base, tetramethylammonium hydroxide (TMAOH), as etchant.^[^ ^20^ ^]^ The Cl‐terminated MXenes including Ti_3_C_2_Cl_2_ and Ti_2_CCl_2_ can be achieved by the reaction of the strong Lewis acidic molten salts ZnCl_2_ with Ti_3_ZnC_2_ and Ti_2_ZnC, respectively (Figure 2c).^[^ ^40^ ^]^ Besides, a redox‐controlled A‐site Lewis acidic etching method to synthesize MXenes from Si‐, Zn‐, and Ga‐based MAX precursors was recently proposed.^[^ ^41^ ^]^ First, the Ti_3_SiC_2_ MAX phase was immersed in CuCl_2_ Lewis molten salt at 750 °C. Then the reaction between them led to the formation of Ti_3_C_3_T*_x_*. The final MXene material was obtained by immersing into ammonium persulfate to remove Cu particles from surface.

### 2.3 Increasing the Yield of Single‐/Few‐Layer MXenes

So far, many strategies have been proposed to increase the delamination yield of single‐/few‐layer MXenes including intercalation, sonication, manual shaking, and so on. Clearly, organic molecules and water can be used as intercalation agents to cleave nanosheets from multilayer MXenes. The stacked Ti_3_C_2_ layers could be successfully delaminated into single layer through intercalation of dimethyl sulfoxide (DMSO).^[^ ^42^ ^]^ Nb_2_CT*_x_* has been delaminated by inserting isopropylamine (i‐PrA) between layers followed by mild sonication in water (Figure 2d).^[^ ^26^ ^]^ Moreover, using organic base including TBAOH, choline hydroxide and n‐butylamine followed by agitation or mild sonication in water to delaminate multilayered MXenes resulted in the large‐scale delamination of layers.^[^ ^43^ ^]^ The yield of small MXenes can be increased to 81.4% through the water freezing‐and‐thawing (FAT) strategy combining with sonication (Figure 2e).^[^ ^44^ ^]^ The space between the adjacent MXene layers became larger due to the intercalation of water molecules, revealing that the expansion force could promote the exfoliation.

## 3 Structures and Electronic Properties of MXenes

**Figure 3:**
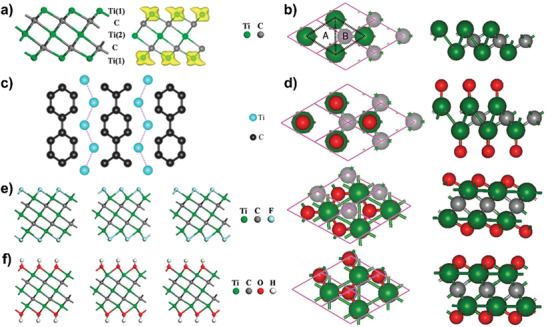
a) Side view of the bare Ti_3_C_2_ monolayer composed of a quintuple layer with Ti(1)‐C‐Ti(2)‐C‐Ti(1) stacking modes (left) and the computed spin density distribution (right). Reproduced with permission.^[^ ^45^ ^]^ Copyright 2012, American Chemical Society. b) Top and side views of the obtained 2D M_2_X layer. The dotted lines exhibit the A‐type and B‐type hollow sites described in the text. Reproduced with permission.^[^ ^46^ ^]^ Copyright 2013, WILE‐VCH GmbH. c) Zigzag Ti atom chain and n‐biphenyl structural unit in the TiC_3_ monolayer. Reproduced with permission.^[^ ^47^ ^]^ Copyright 2018, American Chemical Society. d) Top and side views of models of the functionalized MXene systems (Model 1, Model 2, and Model 4 from top to down. The faces of Model 3 resemble models 2 and 4). Green, gray, and red balls show positions of transitional metals, the carbon/nitrogen atoms, and the attached functionalized groups, respectively. Reproduced with permission.^[^ ^46^ ^]^ Copyright 2013, WILE‐VCH GmbH. e) Side views of I‐Ti_3_C_2_F_2_, II‐Ti_3_C_2_F_2_, and III‐Ti_3_C_2_F_2_ from left to right and f) I‐Ti_3_C_2_(OH)_2_, II‐Ti_3_C_2_(OH)_2_, and III‐Ti_3_C_2_(OH)_2_ from left to right. Reproduced with permission.^[^ ^45^ ^]^ Copyright 2012, American Chemical Society.

The bare monolayer MXene generally possesses a hexagonal‐like unit cell. Typically, M_3_X_2_ system contains quintuple layers stacked in a sequence of M‐X‐M‐X‐M, which can be seen as three M atomic layers intercalated with two X atomic layers (**Figure** **3**a), forming an edge‐shared M_6_X octahedron.^[^ ^45^ ^]^ In the M_2_X system, the X layer is sandwiched between the M bilayer (Figure 3b), forming an edge‐shared M_6_X octahedron.^[^ ^46^ ^]^ Specifically, the existence of n‐biphenyl was observed in TiC_3_ MXenes (Figure 3c), which not only minimizes the energy of monolayer but also endows more C atoms presented on the surface.^[^ ^47^ ^]^ In addition, owing to the fact that the coordination number of one M ion is usually six, it has been inferred that there are six chemical bonds of M—X and surface chemical groups, leading to the production of M_2_XF_2_, M_2_XO_2_, and M_2_X(OH)_2_. As shown in Figure 3b, two types of hollow sites exist on the surface of M_2_X, where no X atom is available for hollow sites A and an X atom is available for hollow sites B. Therefore, four possible configurations for surface groups of M_2_X system have been proposed (Figure 3d), in which two surface groups on the top of two M atoms for Model 1, two surface groups on top of hollow sites A for Model 2, one surface group on top of hollow site A and the other surface group on top of hollow site B for Model 3, and two surface groups on the top of hollow sites B for Model 4.^[^ ^46^ ^]^ Meanwhile, DFT calculations indicated that the stability of M_2_X system decreases in the order of ‐OH, ‐O, and ‐F terminations, suggesting that more favorable ‐O and ‐OH surface groups would likely to substitute ‐F surface groups.^[^ ^48^ ^]^

More importantly, the electronic properties of MXenes are strongly related with the terminal surface groups generated during the synthetic processes.^[^ ^20^, ^49^, ^50^, ^51^, ^52^ ^]^ According to the density of states (DOS) calculated by DFT, most bare MXenes are intrinsic metallic and have high density of carries, suggesting their good electronic conductivity. However, when terminated with ‐F, ‐O, or ‐OH surface groups, the resultant monolayers can be changed to semiconductors. For example, the metallic Lu_2_C could transform into the semiconductor after the functionalization of ‐F and ‐OH groups.^[^ ^52^ ^]^ Since ‐F and ‐OH surface groups may decrease the ionic conductivity and impede the ion transport, it is believed that decreasing the concentration of them could improve the electrochemical properties of MXene‐based materials, while both the bare and O‐terminated MXenes are highly desirable for the electrode materials.

The spatial arrangements of MXenes also have great effect on the electronic properties. As shown in Figure 3e, I‐Ti_3_C_2_F_2_ and III‐Ti_3_C_2_F_2_ show semiconducting states with the bandgap of 0.04 and 0.03 eV, respectively, while II‐Ti_3_C_2_F_2_ is a metal. In addition, I‐Ti_3_C_2_(OH)_2_ and III‐Ti_3_C_2_(OH)_2_ have semiconductor characters possessing the bandgap of 0.05 and 0.07 eV, respectively, while II‐Ti_3_C_2_(OH)_2_ is metallic (Figure 3f).^[^ ^45^ ^]^ Meng et al. also reported that Zr_2_CO_2_ MXene is semiconductor with a bandgap of 0.92 eV, while Zr_3_C_2_O_2_ still is metallic.^[^ ^53^ ^]^

## 4 Sodium‐Ion Intercalation Chemistries in MXenes

**Figure 4:**
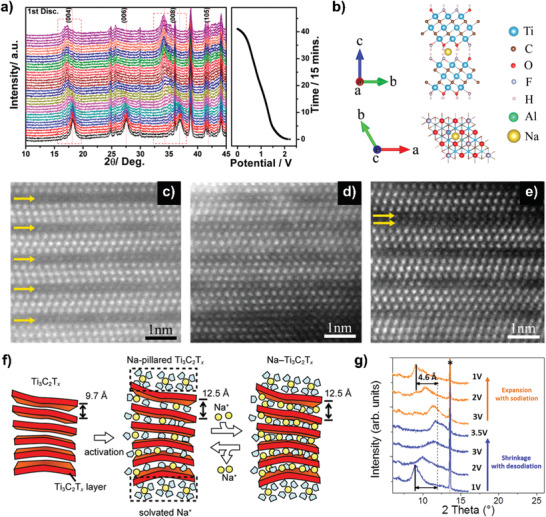
a) In situ XRD patterns of Ti_3_C_2_T*_x_* during electrochemical intercalation of the Na ions, b) optimized geometries of Na*_x_*Ti_3_C_2_T*_x_* from side and top view, and c–e) high‐angle annular dark‐field imaging images of Ti_3_C_2_T*_x_* electrodes upon Na intercalation with cutoff potential of 0.5 V. Reproduced with permission.^[^ ^55^ ^]^ Copyright 2015, American Chemical Society. f) Schematic illustration for the proposed mechanism of Na^+^ insertion into Ti_3_C_2_T*_x_*. Reproduced with permission.^[^ ^56^ ^]^ Copyright 2016, American Chemical Society. g) XRD patterns of V_2_CT*_x_* at different potentials. Reproduced with permission.^[^ ^57^ ^]^ Copyright 2015, American Chemical Society.

Intercalation chemistry plays a vital role in the energy conversion and storage, especially in the capacitors, where the ion intercalation/deintercalation intrinsically contributes to the capacitance. In the aqueous electrolytes, sodium ions can be spontaneously intercalated into Ti_3_C_2_T*_x_* layers.^[^ ^54^ ^]^ By contrast, in the nonaqueous electrolytes, sodium ions are not spontaneously but electrochemically intercalated into MXene sheets.^[^ ^13^ ^]^ Besides, in the combination of scanning transmission electron microscope and in situ X‐ray diffraction (XRD), the sodium‐ions intercalation chemistry in T_3_C_2_T*_x_* has been clarified.^[^ ^55^ ^]^ The XRD patterns (**Figure** **4**a) indicated that sodium ions are intercalated into Ti_3_C_2_T*_x_* reversibly via two‐phase transition and solid‐solution reactions. The intercalated sodium ions prefer to exist on the top of the C atoms rather than the Ti atoms of Ti_3_C_2_ monolayer (Figure 4b). These intercalated sodium ions are intercalated at the surface and then diffuse into the bulk, which partially (Figure 4c) or fully (Figure 4d) occupy the interlayers of Ti_3_C_2_T*_x_*. Meanwhile, the double sodium atomic layers are formed (Figure 4e) in a monolayer upon extensive sodium intercalation, which share the same site. In addition, through solid‐state ^23^Na magic angle spinning NMR and DFT calculations, the reversible sodium intercalation/deintercalation into the interlayer space of Ti_3_C_2_T*_x_* occurred in a nonaqueous electrolytes has been also revealed.^[^ ^56^ ^]^ As shown in Figure 4f, the interlayer distance is first expanded during the first sodiation process due to the solvent molecules and desolvated sodium‐ions intercalation. Then the reversible intercalation/deintercalation of desolvated sodium ions proceeds, while the interlayer distance keeps constant because of the solvent molecule swelling and the trapped sodium‐ions pillaring.

Recently, the studies about the mechanism of sodium intercalation in V_2_CT*_x_* show that there is a 0.23 nm expansion or shrinkage during the reversible intercalation and deintercalation (Figure 4g), indicating that two sodium layers could be intercalated.^[^ ^57^ ^]^ The sodium‐ions behavior in Ti_2_C MXene sheets terminated with ‐O groups was also investigated. The results demonstrated that a large quantity of sodium could be intercalated into the Ti_4_C_2_O_4_ sheets, while the overall structures of MXenes are maintained, assuring the stable structure of Na*_x_*Ti_4_C_2_O_4_ (8 ≤ *x* ≤ 12) during the severe charging–discharging process.^[^ ^58^ ^]^ When more than two sodium layers are intercalated, the inner sodium will be bonded to their neighbors by weaker metallic bonding, combining with the outer sodium layer shielding the O^2−^ anions. Moreover, there is a continuous expansion in the spacing of MXene sheets with the increasing sodium content.

## 5 MXene‐Based Materials for Sodium‐Ion Storage

By theoretical simulations and experimental measurements, the application of MXene‐based materials in the sodium‐ion storage has been widely investigated. The results demonstrate that MXene‐based materials show great potential as promising hosts for sodium‐ion storage. They also provide profound insight on the sodium‐ion storage mechanisms for MXene‐based materials. Owing to the facts that the properties of MXenes can be tuned by the structures such as interlayer spacing and surface groups, numerous efforts have been made in MXene‐based materials to optimize the electrochemical performance. The association of structures with performances as well as the sodium‐ion storage mechanisms of MXene‐based materials is clearly discussed in this section, which will provide an overview of MXene‐based materials for sodium‐ion storage.

### 5.1 MXene Monolayer as an Anode Material for SIBs Predicted by Theoretical Calculations

The inherent metallicity and unique geometry structure of MXenes inspire many researchers to explore their potential as anodes for SIBs. To date, many studies have been systematically conducted on the adsorption and diffusion behaviors for sodium ions on the surface of MXenes monolayer using first‐principles DFT calculations and ab initio molecular dynamics simulations. For example, Yu et al. reported a remarkably high storage capacity of 1278 and 1341 mAh g^−1^ for bare and O‐functionalized TiC_3_ monolayer, respectively.^[^ ^47^ ^]^ These high values are attributed to the distinct n‐biphenyl units in the TiC_3_, which provide the large adsorption area, strong sodium‐ions adsorption ability, and low barrier energy, making the TiC_3_ monolayer as an ideal anode material for SIBs. Besides, other types of MXenes, such as Sc_2_C,^[^ ^59^ ^]^ o‐ScC_2_,^[^ ^51^ ^]^ o‐ScN_2_,^[^ ^51^ ^]^ MoC_2_,^[^ ^60^ ^]^ V_3_C_2_,^[^ ^61^ ^]^ Mn_2_C,^[^ ^62^ ^]^ MnC,^[^ ^63^ ^]^ monolayer also have been predicted as the promising anode candidates for SIBs due to their low sodium‐ions diffusion energy barrier and high theoretical capacity.

The important characteristics determining the performance of electrode materials including adsorption energy, diffusion energy barriers, average OCVs, and sodium storage capacity predicted by theoretical calculations are summarized in **Table** **1**. As can be seen, the negative adsorption energies reveal that sodium can be spontaneously adsorbed on the surface of MXenes. In fact, there are shallow and deep adsorption sites in the interlayer of MXenes, suggesting that these electrodes can not only store quite large quantity of charge but have relatively rapid discharge rate.^[^ ^64^ ^]^ Sodium ions prefer to absorb on the hollow sites of M_2_CO_2_ layers, resulting in the sodiated structure and chemical formula of M_2_CO_2_Na_2_.^[^ ^65^ ^]^ The average voltage is significantly related with the distance between the transition metal layers and the adsorbed sodium ions, which mainly influences the interaction strength between them and the electrostatic energies of system. Meng et al. found that the OCV of Zr_2_CO_2_ and Zr_3_C_2_O_2_ decrease monotonously with the increasing of the number of electrons involved in the electrochemical process, and the OCV for Zr_2_CO_2_ is lower than that for Zr_3_C_2_O_2_ MXene.^[^ ^53^ ^]^ Besides, the Zr_3_C_2_O_2_ and Zr_2_CO_2_ could accommodate up to two layers of sodium ions, leading to the high capacities of 326 and 474 mAh g^−1^, respectively.

**Table 1:** Comparison of the sodium‐ion adsorption energy (*E*_ads_) for the first layer, diffusion barrier, average open‐circuit voltage (OCV, vs Na/Na^+^), and specific capacity of different MXenes monolayer as anode materials for SIBs predicted by theoretical calculations

MXenes	Terminated group	*E*_ads_ [eV atom^−1^]	Diffusion barrier [eV]	OCV [V]	Specific capacity [mAh g^−1^]	Ref.
TiC_3_	bare	−0.50	0.18	0.18	1278	^[^ ^47^ ^]^
Ti_2_C	bare	−0.79	0.021		348.70	^[^ ^50^ ^]^
	‐C	−2.54	0.155		301.58	
	‐O	−1.45	0.059		288.62	
	‐S	−1.26	0.095		246.07	
Ti_3_C_2_	bare	−0.262	0.10	0.14	351.8	^[^ ^67^, ^71^, ^72^ ^]^
	‐O	−0.829	0.22		250	
	‐S	−2.11	0.11		463	
Ti_3_N_2_	bare	−1.0	0.041	0.51	312	^[^ ^66^ ^]^
	‐F	−0.5	0.180	0.06	85	
	‐O	−2.0	0.181	0.721	258	
	‐OH	−0.1				
V_2_C	bare	−0.528	0.01	0.82	470.65	^[^ ^65^, ^68^, ^71^ ^]^
	‐O	−0.876	0.15	0.52	367.41	
	‐S	−1.26	0.06	0.49	301.12	
V_3_C_2_	bare	−1.24	0.02		606.42	^[^ ^61^ ^]^
	‐O	−2.73	0.31		513.5	
Nb_2_C	bare	−0.574			252	^[^ ^71^ ^]^
	‐O	−0.665			194	
Cr_2_C	‐O		0.09	0.26	276	^[^ ^65^, ^73^ ^]^
MnC	bare	−2.83	0.174		475	^[^ ^63^ ^]^
Mn_2_C	bare	−0.44	0.022	0.25	443.6	^[^ ^62^, ^65^ ^]^
	‐O		0.15	0.80		
MoC	bare	−0.89		0.80	248.2	^[^ ^60^ ^]^
MoC_2_	bare	−1.76	0.23	0.28	446.9	^[^ ^60^ ^]^
Mo_2_C	bare	−1.01		0.31	262.9	^[^ ^60^, ^65^ ^]^
	‐O		0.14	0.19		
Sr_2_C	bare	−0.61	0.012	0.24	362	^[^ ^59^ ^]^
o‐SrC_2_	bare	−0.28	0.050	0.08	777	^[^ ^51^ ^]^
o‐SrN_2_	bare	−0.75	0.269	0.10	735	^[^ ^51^ ^]^
Zr_2_C	bare	−0.77	0.03			^[^ ^53^ ^]^
	‐O	−0.81	0.29		474	
Zr_3_C_2_	bare	−0.79	0.03			^[^ ^53^ ^]^
	‐O	−1.56	0.32		326	
Hf_3_C_2_	bare	−1.91	0.018	0.46	444.9	^[^ ^74^ ^]^
	‐F	−0.91	0.083	1.60		
	‐O	−2.93	0.231	0.46		
	‐OH	−0.94	0.013	3.11		
MoCrC_2_	bare	−0.28	0.027	0.89	297.91	^[^ ^75^ ^]^

Furthermore, much efforts have been made on the effects of surface groups of MXenes on the sodium‐ion storage performance. The presence of functional groups (‐F, ‐OH) of Ti_3_N_2_ MXene is unfavorable to sodium‐ions migration and decreases theoretical capacity except for ‐O groups.^[^ ^66^ ^]^ By contrast, the multilayered adsorption ability for sodium of Ti_3_C_2_S_2_ monolayer has been demonstrated and the achieved theoretical capacity of 463 mAh g^−1^ is larger than that of bare and O‐functionalized Ti_3_C_2_ monolayer.^[^ ^67^ ^]^ Meanwhile, the OCVs for sodium follow the order of bare Ti_3_C_2_ < Ti_3_C_2_S_2_ < Ti_3_C_2_O_2_. The adsorption energy of sodium for Ti_2_C MXenes follow the order of bare < S < O < C, suggesting that C‐terminated Ti_2_C prefers to absorb sodium ions than others.^[^ ^50^ ^]^ The theoretical capacity of Ti_2_CC_2_ is higher than that of O‐ and S‐terminated Ti_2_C MXenes but lower than that of the bare Ti_2_C. In addition, the diffusion energy barriers follow the order of bare < O < S < C. These results reveal that sodium ions could freely and easily migrate on the bare MXene monolayer, whereas the terminated groups tend to impede the sodium‐ions diffusion.^[^ ^68^ ^]^

The interlayer spacing also plays an important role in determining the capacities of rechargeable batteries. The stable multilayer sodium adsorption can be obtained on the bare and O‐terminated Ti_3_C_2_ MXenes with enlarged interlayer distance of 7 nm.^[^ ^69^ ^]^ The calculated diffusion energy barriers on bare, OH‐, F‐, and O‐terminated Ti_3_C_2_ MXenes are 0.02, 0.013, 0.19, and 0.20 eV, respectively, indicating that sodium ions can easily migrate on the interlayer‐expanded Ti_3_C_2_ MXenes. Moreover, according to the calculated theoretical storage capacities, the interlayer‐expansion approach could improve the sodium storage capacities of MXene‐based SIBs.

To get more insight into the interaction between the surfaces of MXenes and sodium ions, many works have been carried out on the electronic structures of system before and after sodium adsorption. The sodium‐ion adsorption has significant impact on the electronic transport of Ti_3_C_2_ with ‐O groups, whereas the effects become less obvious in bare, F‐, and OH‐terminated systems.^[^ ^70^ ^]^ This phenomenon stems from the localization of electronic states and is strongly associated with the surface chemistry. The Fermi level (*E*_f_) for the bare, F‐, and O‐terminated Ti_3_C_2_ MXenes shift upward while the OH‐functionalized nanosheets shift downward, suggesting the existence of strong electronic interaction between the surfaces of MXenes and the adsorbed sodium atoms.^[^ ^69^ ^]^ The sodium adsorption also made *E*_f_ upward shift in the Zr_2_CO_2_ MXene, making the semiconductor Zr_2_CO_2_ MXene switch to a metal and then ensuring the fast electron transport in the electrode for SIBs.^[^ ^53^ ^]^

Additionally, Ti_2_CO_2_/graphene and V_2_CO_2_/graphene heterostructures are encouraging in the application of SIBs due to the low diffusion energy barriers for sodium and estimated high capacities.^[^ ^76^ ^]^ The heterostructures composed of Ti_2_CT_2_ (T = F, O) and MoS_2_ exhibit more negative adsorption energies and larger electrical conductivities than the pure components monolayer.^[^ ^77^ ^]^ The diffusion barrier for sodium and theoretical capacity for SIBs are calculated as 0.37 eV and over 430 mAh g^−1^, respectively. Furthermore, the excellent mechanical flexibility and large ultimate tensile strains are favorable to the application of flexible batteries. Tang et al.^[^ ^18^ ^]^ constructed 16 different heterostructures of bare or O‐terminated Ti, V, Nb or Mo‐based MXenes with MoS_2_ or VS_2_. By first principles calculations, they found that sodium ions could be intercalated into the interlayer of structures containing O‐terminated MXenes, whereas the intercalation of sodium ions in the structures with bare MXenes is energy unfavorable. Meanwhile, only the structures of O‐terminated MXenes with VS_2_ allow five layers of sodium ions, while the others have the distortion. Furthermore, the diffusion barriers for sodium ions of the first layer and interlayer and OCVs of these heterostructures are 0.086–0.221 eV, 0.0002–0.033 eV, and 0.16–0.36 V, respectively, making them promising candidates for SIBs. Besides, the ultralow overpotential for *η*_ORR_/*η*_OER_ of Ti_2_CO_2_/VS_2_ heterostructure proved that it also has a great potential in Na‐O_2_ batteries.

### 5.2 Pure MXenes for Sodium‐Ion Storage

Like most of 2D materials, MXenes suffer from the tendency to stack during the experiment, which limits the penetration of electrolyte and impedes the ionic transport. Therefore, in this section, three main strategies to make full use of their electrochemical performance are summarized including 1) synthesizing single‐/few‐layer MXenes, 2) increasing the interlayer spacing of MXenes by introducing intercalation agents, and 3) creating 3D porous structures. **Table** **2** lists the performance of pure MXenes for sodium‐ion storage in recent years, including the compositions/configurations and their corresponding long‐term and rate capacities.

**Table 2:** Comparison of pure MXenes for sodium‐ion storage

Classification	Material	Device	Long‐term capacity	Cycle number	Current density	Rate capability	Ref.
Multilayer	Ti_3_C_2_T*_x_*	SIBs	68.3 mAh g^−1^	1000	0.2 A g^−1^	53.7 mAh g^−1^ at 0.8 A g^−1^	^[^ ^55^ ^]^
		SICs	70 mAh g^−1^	450	0.1 A g^−1^	24 mAh g^−1^ at 5 A g^−1^	^[^ ^79^ ^]^
		SSBs	150 mAh g^−1^	300	0.1 A g^−1^	120.0 mAh g^−1^ at 1 A g^−1^	^[^ ^17^ ^]^
	Ti_3_CN	SIBs	73.5 mAh g^−1^	500	0.2 A g^−1^	98.9 mAh g^−1^ at 0.5 A g^−1^	^[^ ^78^ ^]^
	Ti_2_CT*_x_*	SICs	103 mAh g^−1^	100	0.6 A g^−1^	40 mAh g^−1^ at 5 A g^−1^	^[^ ^13^ ^]^
	V_2_CT*_x_*	SICs	22 mAh g^−1^	300	20 C	70 mAh g^−1^ at 3C	^[^ ^57^ ^]^
	Nb_4_C_3_T*_x_*	SIBs	69 mAh g^−1^	100	0.1 A g^−1^	71.5 mAh g^−1^ at 2 A g^−1^	^[^ ^80^ ^]^
Single‐/few‐layer	Hf_3_C_2_T*_x_*	SICs	47 mAh g^−1^	200	0.2 A g^−1^	29 mAh g^−1^ at 1 A g^−1^	^[^ ^15^ ^]^
	f‐Ti_3_C_2_T*_x_*‐milled	SIBs	76 mAh g^−1^	1500	1 A g^−1^	110 mAh g^−1^ at 2 A g^−1^	^[^ ^81^ ^]^
Expanded interlayer spacing	Na‐Ti_3_C_2_	SIBs	175 mAh g^−1^	200	0.1 A g^−1^	85 mAh g^−1^ at 2 A g^−1^	^[^ ^84^ ^]^
	Sulfur‐decorated Ti_3_C_2_	SIBs	135 mAh g^−1^	1000	2 A g^−1^	136.6 mAh g^−1^ at 5 A g^−1^	^[^ ^85^ ^]^
	S‐doped Ti_3_C_2_T*_x_*	SIBs	138.2 mAh g^−1^	2000	0.5 A g^−1^	113.9 mAh g^−1^ at 4 A g^−1^	^[^ ^86^ ^]^
	V_2_C@Mn	SIBs	297 mAh g^−1^	1200	0.05 A g^−1^	56 mAh g^−1^ at 5 A g^−1^	^[^ ^87^ ^]^
	CT‐S@Ti_3_C_2_‐450	SIBs	492 mAh g^−1^	100	0.1 A g^−1^	120 mAh g^−1^ at 15 A g^−1^	^[^ ^16^ ^]^
		SICs	122 F g^−1^	10 000	2 A g^−1^	138.5 F g^−1^ at 4 A g^−1^	
	CT‐Sn(II)@Ti_3_C_2_	SIBs	95 mAh g^−1^	200	1 C	100 mAh g^−1^ at 2 C	^[^ ^88^ ^]^
3D porous structure	a‐Ti_3_C_2_T*_x_*	SIBs	50 mAh g^−1^	500	0.2 A g^−1^		^[^ ^89^ ^]^
	Na‐c‐Ti_3_C_2_T*_x_*	SIBs	130 mAh g^−1^	500	0.1 A g^−1^	61 mAh g^−1^ at 1 A g^−1^	^[^ ^90^ ^]^
	c‐Ti_3_C_2_T*_x_*	SIBs	246 mAh g^−1^	50	0.02 A g^−1^	120 mAh g^−1^ at 0.5 A g^−1^	^[^ ^91^ ^]^
	p‐Ti_3_C_2_T*_x_*	SIBs	189 mAh g^−1^	1000	1 A g^−1^	123 mAh g^−1^ at 10 A g^−1^	^[^ ^92^ ^]^
	S‐Ti_3_C_2_T*_x_*	SSBs	577 mAh g^−1^	500	2 C	610 mAh g^−1^ at 5 C	^[^ ^93^ ^]^
	Ti_3_C_2_T*_x_*	SIBs	295 mAh g^−1^	1000	2.5 C	330 mAh g^−1^ at 0.25 C	^[^ ^94^ ^]^
	V_2_CT*_x_*	SIBs	310 mAh g^−1^	1000	2.5 C	340 mAh g^−1^ at 0.25 C	
	Mo_2_CT*_x_*	SIBs	290 mAh g^−1^	1000	2.5 C	370 mAh g^−1^ at 0.25 C	

#### 5.2.1 Multilayer MXenes

**Figure 5:**
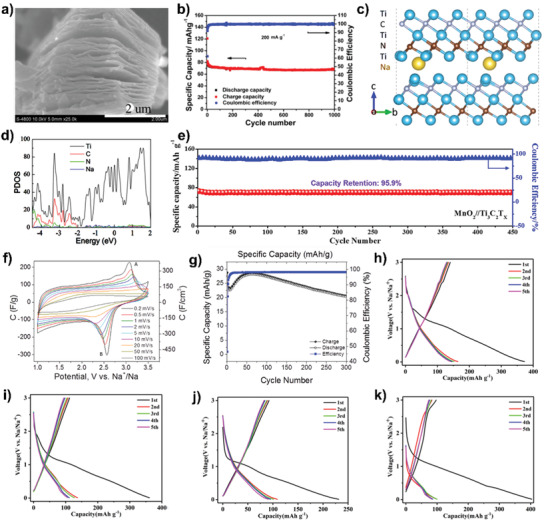
a) SEM images of Ti_3_C_2_T*_x_* and b) long‐term cycling stability of Ti_3_C_2_T*_x_* for SIBs. Reproduced with permission.^[^ ^55^ ^]^ Copyright 2015, American Chemical Society. c) Optimized geometry of layered Ti_3_CN intercalated with Na ions and d) DFT calculated projected density of states (PDOS) of Ti_3_CN. The Fermi level is set to zero. Reproduced with permission.^[^ ^78^ ^]^ Copyright 2018, American Chemical Society. e) Cycling performance of the MnO_2_//Ti_3_C_2_T*_x_* capacitor battery. Reproduced with permission.^[^ ^79^ ^]^ Copyright 2017, Wiley‐VCH GmbH. f) Cyclic voltammetry of V_2_CT*_x_* at different scan rate and g) capacity versus cycle number. Reproduced with permission.^[^ ^57^ ^]^ Copyright 2015, American Chemical Society. h–k) The discharge/charge curves of h,i) Nb_4_C_3_T*_x_*, j) Nb_3.5_Ta_0.5_C_3_T*_x_*, and k) Nb_3.9_W_0.1_C_3_T*_x_* samples at 100 mA g^−1^. Reproduced with permission.^[^ ^80^ ^]^ Copyright 2018, Elsevier Ltd. and Techna Group S.r.l.

Usually, selectively removing the A layer from layer‐structured MAX gives birth to the multilayer‐stacked MXene nanosheets (**Figure** **5**a).^[^ ^55^ ^]^ The Ti_3_C_2_T*_x_* MXenes and Ti_3_AlC_2_ used for SIBs showed the reversible capacity of 100 and 16.8 mAh g^−1^, respectively. The enhancement can be credited to the expanded interlayer spacing due to the removal of Al layer and the increased active storage sites. Meanwhile, at a current density of 0.8 or 0.2 A g^−1^ after 1000 cycles, a reversible capacity of 53.7 or 68.3 mAh g^−1^ could be obtained (Figure 5b). Ti_3_CN MXenes were also used as anodes in SIBs.^[^ ^78^ ^]^ When measured at 0.5 A g^−1^, the capacity of 98.9 mAh g^−1^ was acquired, which was 1.65 times of that pristine Ti_3_C_2_. The improvement was attributed to that introducing more electronegative N atoms into Ti_3_C could increase the electron density of MXenes. The optimized geometry of Ti_3_CN intercalated with sodium ions and the DOS of Ti_3_CN calculated by DFT are shown in Figure 5c,d, respectively. In addition, Ti_3_C_2_T*_x_* MXenes can be used as electrode materials for SICs.^[^ ^79^ ^]^ Assembled with MnO_2_ and Na_2_SO_4_ electrolyte, the capacitor exhibited the reversible capacitance of 24 mAh g^−1^ at 5 A g^−1^ and 70 mAh g^−1^ after 450 cycles at 0.1 A g^−1^ (Figure 5e). When used as the cathode material for SSBs, Ti_3_C_2_T*_x_*@S showed the capacity of 120.0 mAh g^−1^ at 1 A g^−1^ and 150.0 mAh g^−1^ after 300 cycles at 0.1 A g^−1^.^[^ ^17^ ^]^

In general, the mechanisms of double layer in capacitors and ion intercalation/deintercalation in batteries intrinsically lead to the competition between power and energy densities for electrochemical storage. Thus, developing the new electrodes based on pseudocapacitive charge storage mechanism is receiving tremendous attention. The energy storage mechanism for V_2_CT*_x_* MXenes has been investigated by XRD and electrochemical impedance spectroscopy (EIS), which showed the continuous intercalation of sodium ions between layers and various charge‐transfer resistance at different potentials.^[^ ^57^ ^]^ The results demonstrated that both pseudocapacitive and diffusion capacitive occur. The rectangular shape of cyclic voltammetry (CV) curves (Figure 5f) further confirmed the pseudocapacitive behavior. When used as positive electrode for SICs, the full cells obtained a good capacity of 70 mAh g^−1^ at 3 C. After 300 cycles, the capacity was maintained 70% (Figure 5g). The Ti_2_CT*_x_* MXenes also showed the pseudocapacitor behavior with no obvious structural changes, resulting in the excellent efficiency and cycle stability.^[^ ^13^ ^]^ In addition, the study about three types of MXenes including Nb_3.5_Ta_0.5_C_3_T*_x_*, Nb_3.9_W_0.1_C_3_T*_x_*, and Nb_4_C_3_T*_x_* for electrochemical performance toward SIBs (Figure 5h–k) revealed that the substitution of Nb with some Ta or W was inferior for the sodium‐ion storage.^[^ ^80^ ^]^

#### 5.2.2 Single‐/Few‐Layer MXenes

**Figure 6:**
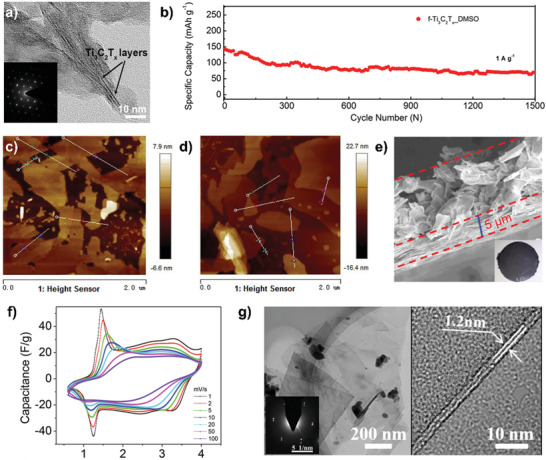
a) TEM images of f‐Ti_3_C_2_T*_x_*_DMSO (inset in part is selective area electron diffraction (SAED)) and b) ultralong cycle life of 1500 cycles achieved by f‐Ti_3_C_2_T*_x_*_DMSO at a current rate of 1 A g^−1^. Reproduced with permission.^[^ ^81^ ^]^ Copyright 2017, American Chemical Society. c,d) Atomic force microscopy images of d‐D‐Ti_3_C_2_T*_x_* and d‐a‐Ti_3_C_2_T*_x_*, respectively. Reproduced with permission.^[^ ^82^ ^]^ Copyright 2018, Elsevier B.V. e,f) Cross‐sectional SEM shows multilayered Ti_3_C_2_T*_x_*/d‐Ti_3_C_2_T*_x_*; inset shows e) the 4 cm diameter bistacked MXene film and f) CV curves of hybrid Na‐ion capacitor at different scan rates. Reproduced with permission.^[^ ^83^ ^]^ Copyright 2018, American Chemical Society. g) Typical bright‐field TEM images of the delaminated (left) and few‐layered (right) Hf_3_C_2_T*_x_* flakes. Reproduced with permission.^[^ ^15^ ^]^ Copyright 2017, American Chemical Society.

It is generally known that the single‐/few‐layer MXenes are not only favorable to fully utilize the surface and electrolyte penetration but also facilitate fast charge transport and sodium‐ions diffusion. As mentioned above, intercalating agents into the multilayer MXenes is an effective method to increase the yield of single‐/few‐layer MXenes. For instance, through high‐energy mechanical‐milling method assisted with DMSO intercalation, the scale delamination of few‐layer Ti_3_C_2_T*_x_* MXenes (denoted as f‐Ti_3_C_2_T*_x_*_DMSO) was achieved due to the high pressure and temperature, which can be observed in high‐resolution transmission electron microscopy (HRTEM; **Figure** **6**a) with a smaller size and large interspace.^[^ ^81^ ^]^ The SIBs based the few‐layer MXene nanosheets delivered a high reversible capacity of 267 mAh g^−1^ at 0.1 A g^−1^ and 76 mAh g^−1^ after 1500 cycles at 1 A g^−1^ (Figure 6b). Moreover, only intercalation of DMSO or alcohol could produce the few‐layer Ti_3_C_2_T*_x_* (denoted as d‐D‐Ti_3_C_2_T*_x_* and d‐a‐Ti_3_C_2_T*_x_*, respectively) with a uniform height of 4 or 7 nm (Figure 6c,d).^[^ ^82^ ^]^ The results demonstrated that much few‐layer structure of d‐D‐Ti_3_C_2_T*_x_* resulted in the larger specific surface area to enhance the sodium‐ion storage.

As a typical instance in SICs application, the bistacked Ti_3_C_2_T*_x_* (Ti_3_C_2_T*_x_*/d‐Ti_3_C_2_T*_x_*) electrodes with the first layer of delaminated nanosheets with a compact morphology and second layer of multilayer particles with an open structure (Figure 6e) were fabricated and combined with an activated carbon (AC) as cathode.^[^ ^83^ ^]^ The capacitor exhibited an energy density of 39 Wh kg^−1^ at the rate of 1 C and could maintain 60% at the rate of 60 C. Besides, the pair of redox peaks shown in Figure 6f confirmed that the hybrid SIC system possessed the double‐layer and redox mechanisms for sodium storage. Recently, Si‐alloying‐facilitated etching process has been explored to produce few‐layer Hf_3_C_2_T*_x_* MXenes (Figure 6g).^[^ ^15^ ^]^ To understand the underlying mechanisms, first‐principles DFT calculations were carried out. It was found that due to the partial substitution of Al with Si, the bond strength of Hf‐C and the adhesive energy of the etching interface have been weakened, facilitating the etching process. The fabricated SIBs showed the reversible capacity of 47 mAh g^−1^ at a current density of 0.2 A g^−1^ after 200 cycles due to the intercalation of sodium ions rather than the conversion reaction. Besides, the X‐ray photoelectron spectroscopy (XPS) results suggested that a relatively high ratio of ‐O terminations in Hf_3_C_2_T*_x_* are favorable to the capacities. These findings indicate that the delaminated MXenes with 2D structures are strong candidate electrode materials for energy storage, especially for applications where size is important.

#### 5.2.3 MXenes with Expanded Interlayer Spacing

**Figure 7:**
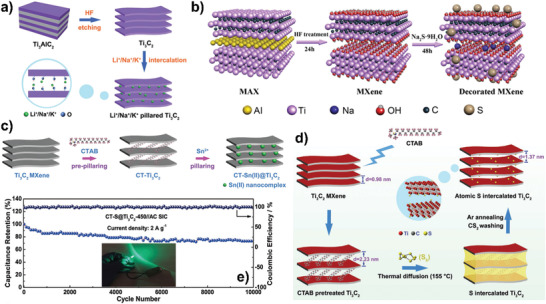
a) A schematic of the fabrication process used to prepare the alkali metal ion pillared Ti_3_C_2_ materials. Reproduced with permission.^[^ ^84^ ^]^ Copyright 2018, Royal Society of Chemistry. b) Schematic illustration of the preparation of sulfur‐decorated Ti_3_C_2_ MXenes. Reproduced with permission.^[^ ^85^ ^]^ Copyright 2019, Elsevier B.V. c) Schematic illustration of preparation of CT‐Sn(II)@Ti_3_C_2_ by CTAB prepillaring process followed by a method of Sn^2+^ pillaring. Reproduced with permission.^[^ ^88^ ^]^ Copyright 2018, WILEY‐VCH GmbH. d,e) Schematic illustration of d) the synthesis of S atoms intercalated Ti_3_C_2_ and e) long‐term cycling performance of the SIC at 2 A g^−1^. Reproduced with permission.^[^ ^16^ ^]^ Copyright 2019, WILEY‐VCH GmbH.

Since the cations and organic agents can be easily intercalated to the nanosheets of MXenes, it has been widely used to enlarge the interlayer space to improve sodium‐ions transport kinetics and increase sodium storage sites. DFT calculations have predicted that the MXenes with enlarged interlayer space could achieve higher performance for sodium‐ions storage.^[^ ^69^ ^]^ Due to the pillaring effect of cation ions such as Li^+^, Na^+^, and K^+^, after immersing Ti_3_C_2_ MXenes into corresponding alkali solutions, the interlayer spacing of MXenes increased to 1.26 nm (**Figure** **7**a).^[^ ^84^ ^]^ The pillaring Na^+^ in the interlayer increased the valence state of Ti, leading to the more reversible redox reactions and clear pseudocapacitance features during sodiation/desodiation processes. The SIBs based on them showed the reversible capacity of 175 mAh g^−1^ after 200 cycles at 0.1 A g^−1^ and 85 mAh g^−1^ after 2000 cycles at 2 A g^−1^. Meanwhile, when used in the SICs combined with AC cathode, the capacity could be retained 78.4% after 15 000 cycles at 2 A g^−1^. Soaking Ti_3_C_2_ MXenes into Na_2_S solution brought about the sulfur‐decorated MXenes with interlayer spacing of 1.27 nm (Figure 7b), constructing the efficient and stable sodium diffusion paths.^[^ ^85^ ^]^ The fabricated SIBs delivered impressive electrochemical performance with 135 mAh g^−1^ after 1000 cycles at 2 A g^−1^ and 136.6 mAh g^−1^ at 5 A g^−1^, which can be attributed to the synergistic effect of decorated sulfur groups and enlarged interlayer spacing and the hybrid storage mechanisms of surface‐controlled and intercalation pseudocapacitance. By simple sulfidation of Ti_3_C_2_T*_x_* with thiourea, Li et al. prepared S‐doped Ti_3_C_2_T*_x_*. The expanded interlayer spacing as well as the high contribution of surface‐controlled capacitance rendered a superior sodium storage performance.^[^ ^86^ ^]^ This work highlighted the efficiency of S doping method and provided a new strategy for rational designing the heteroatom‐doped MXenes. The intercalated Mn^2+^ could not only enlarge the interlayer spacing of V_2_C MXenes but also form a V—O—Mn covalent bond, which effectively inhibiting the structural collapse and rendering a capacity of 297 mAh g^−1^ after 1200 cycles at 0.05 A g^−1^ for SIBs.^[^ ^87^ ^]^

In addition, the pretreatment cetyltrimethylammonium bromide (CTAB) followed by Sn^2+^ pillaring has been proposed to fabricate pillared Ti_3_C_2_ MXene with ultralarge interlayer spacing, which was denoted as CT‐Sn(II)@Ti_3_C_2_ (Figure 7c).^[^ ^88^ ^]^ After pillaring with CTAB and Sn^2+^, the interlayer spacing of Ti_3_C_2_ first increased to 2.2 nm and then decreased to 1.9 nm, suggesting the successful intercalation of CTAB and ion‐exchange interaction between CTA^+^ and Sn^2+^. The CT‐Sn(II)@Ti_3_C_2_ was used as the matrix for sodium metal anode, which accommodated the deposited sodium by pillar effect and effectively guided the nucleation and growth of sodium within the interlayer space, preventing formation of sodium dendrite and benefiting for the uniform sodium deposition. As a consequence, the CT‐Sn(II)@Ti_3_C_2_ electrode endowed a high areal capacity of 5 mAh cm^−2^ after 500 cycles at 10 mA cm^−2^. Subsequently, they introduced S atoms into the interlayer of Ti_3_C_2_ after preintercalation of CTAB with thermal diffusion of elemental S and annealing process (Figure 7d), forming an interlayer‐expanded structure through Ti—S bonding.^[^ ^16^ ^]^ The expanded interlayer spacing with S‐functionalized interface contributed to the incremental storage sites and fast sodium‐ions storage kinetics. When used in the sodium‐based half‐cells, the electrode delivered the best MXene‐based sodium‐ion storage rate performance (531, 468, 413, 358, 304, 223, 120 mAh g^−1^ at 0.1, 0.25, 0.5, 1, 2, 5, and 15 A g^−1^, respectively). The CT‐Ti_3_C_2_ matrix, redox reaction between Na^+^ and S bonds as well as the pillar effect contributes to the enhanced interface‐dominated pseudocapacitance. DFT calculations revealed that the absorption of Na atoms on the Ti_3_C_2_S_2_ and Ti_3_C_2_O_2_ were lower than that of Ti_3_C_2_ and Ti_3_C_2_F_2_ and S‐doped MXenes could accommodate two layers of sodium ions, directing improving the sodium‐ion storage capability. Moreover, the fabricated SICs with AC cathode exhibited excellent long‐term cycling performance with the capacity retention of 73.3% after 10 000 cycles at 2 A g^−1^ with around 100% Coulombic efficiency (CE; Figure 7e).

#### 5.2.4 MXenes with 3D Porous Structure

Assembling the 2D materials into 3D porous structures could prove a promising solution to the problems of poor charge transport in electrode materials, hence leading to the devices with high performance.

**Figure 8:**
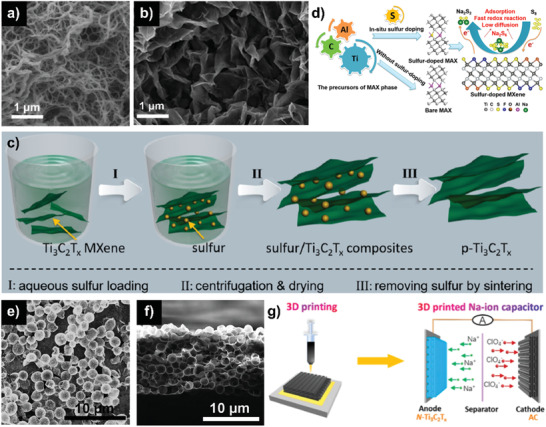
a) SEM images of a‐Ti_3_C_2_ MNRs. Reproduced with permission.^[^ ^89^ ^]^ Copyright 2017, Elsevier Ltd. b) SEM images of Na‐c‐Ti_3_C_2_T*_x_* flocculated networks. Inset shows a higher magnification image. Reproduced with permission.^[^ ^90^ ^]^ Copyright 2018, Royal Society of Chemistry. c) Schematic preparation of p‐Ti_3_C_2_T*_x_*. Reproduced with permission.^[^ ^92^ ^]^ Copyright 2018, American Chemical Society. d) Schematic diagram demonstrating the preparation of sulfur‐doped MXene, along with the discharge process in sulfur‐doped MXene/S cathode, where sodium redox reduction is accelerated and sodium polysulfide shuttling is minimized. Reproduced with permission.^[^ ^93^ ^]^ Copyright 2019, American Chemical Society. e) SEM images of hollow Ti_3_C_2_T*_x_* spheres and f) cross‐sectional SEM images of the 3D macroporous Ti_3_C_2_T*_x_* film. Reproduced with permission.^[^ ^94^ ^]^ Copyright 2017, WILEY‐VCH GmbH. g) Schematic diagram of charging process of N‐Ti_3_C_2_T*_x_*//AC SIC. Reproduced with permission.^[^ ^14^ ^]^ Copyright 2020, American Chemical Society.

In this regard, Lian et al. first reported the fabrication of 3D Ti_3_C_2_ by continuous shaking treatment of HF‐etched Ti_3_C_2_ in alkaline KOH solution (a‐Ti_3_C_2_ MNRs), which not only increasing the interlayer spacing to 1.25 nm but also resulting in the porous frameworks of nanoribbons (**Figure** **8**a).^[^ ^89^ ^]^ Due to the ion exchange and electrostatic interaction, the continuous shaking improved the diffusion of K^+^ and OH^−^, thus splitting the short nanoribbons from the delaminated nanosheets. These alkalized MXenes exhibited good performance for SIBs with a high reversible capacity of 168 mAh g^−1^ at 0.02 A g^−1^. The 3D porous networks of Ti_3_C_2_T*_x_* could be constructed by simply adding alkali into multilayered Ti_3_C_2_T*_x_*, including NaOH, LiOH, KOH, and TBAOH (TBA^+^ represents tetrabutylammonium), creating many irregular large pores with diameters of 100–400 nm (Figure 8b).^[^ ^90^ ^]^ Reducing the pH of Ti_3_C_2_T*_x_* colloidal solution with acid, such as HCl, H_2_SO_4_, and HNO_3_ could also achieve 3D porous open structure of MXenes.^[^ ^91^ ^]^ The addition of H^+^ reduced the negative surface charges, resulting in the rapid aggregation owing to the Van der Waals force and finally complete flocculation and crumpling. Via a sulfur loading‐removal strategy, Xie et al. prepared a porous Ti_3_C_2_T*_x_* (p‐Ti_3_C_2_T*_x_*).^[^ ^92^ ^]^ As shown in Figure 8c, the sulfur was first dissolved in ethlyenediamine (EDA) and then drop added into the Ti_3_C_2_T*_x_* colloidal solution, followed by the addition of HCl to precipitate sulfur on MXene nanosheets and finally heat‐treated at 400 °C to remove sulfur. The resulted open morphology provided the interconnected ion storage reservoirs and improved the electrolyte/electrode interfacial interaction, which simultaneously ensuring the fast electron transfer and the efficient ionic transport. Thus, good capability of 124 mAh g^−1^ at 10 A g^−1^ and cycling stability for 1000 cycles of SIBs were achieved. According to the CV analysis, the predominate nondiffusion‐limited charge storage mechanism together with the high conductivity of MXenes afforded the ultrafast sodium‐ion storage, overcoming the trade‐off between the energy and power densities. Interestingly, the wrinkled structure with enlarged layers of Ti_3_C_2_T*_x_* can be achieved by employing a mixture of S and Al during preparing the MAX phase rather than pure Al (Figure 8d) followed by etching with LiF‐HCl and freeze‐drying process.^[^ ^93^ ^]^ When applied as an electrode host in SSBs, the matrix delivered high polarity with sodium polysulfides, restricting the diffusion of sodium polysulfides. As a result, the devices showed a reversible capacity of 577 mAh g^−1^ after 500 cycles at 2 C. DFT results further demonstrated that sodium polysulfide molecules preferred to bind to the surface of S‐Ti_3_C_2_T*_x_* rather than O‐Ti_3_C_2_T*_x_* or F‐Ti_3_C_2_T*_x_*. This suggested that the incorporation of sulfur terminations could greatly facilitate the redox reactivity, resulting in the higher rate capability of Na–S batteries.

What is more, 3D porous structures of MXenes have been produced by facile templating route. Zhao et al. successfully fabricated Ti_3_C_2_T*_x_* hollow spheres (Figure 8e) through sacrificial poly(methyl methacrylate) (PMMA) spherical templates.^[^ ^94^ ^]^ Due to the interaction between the surface groups, the surface of PMMA spheres was spontaneously wrapped by MXenes nanosheets. After thermal evaporation, hollow MXene spheres formed, resulting in the 3D porous architecture of electrode (Figure 8f) through vacuum‐filtering. Based on this method, they also obtained 3D porous V_2_CT*_x_* and Mo_2_CT*_x_* film electrode. When used as anodes in SIBs, V_2_CT*_x_* exhibited best rate and cycling performance among the three 3D electrodes due to the largest interlayer spacing. It is noteworthy that a nondiffusion‐limited and pseudocapacitive mechanism of sodium‐ion storage at the surface of MXenes was also confirmed by CV profiles, rendering a higher sodium‐ion capacity than the electrodes based on the double‐layer mechanism and a higher rate capability relative to the bulk Faradic electrodes. Fan et al. reported the crumpled nitrogen‐doped porous Ti_3_C_2_T*_x_* (N‐Ti_3_C_2_T*_x_*) via sacrificial melamine formaldehyde (MF) nanospheres templates.^[^ ^14^ ^]^ The surface modification of nitrogen atoms was favorable to the electrical conductivity of MXenes and redox reactivity. The interconnected nanosheets with porous framework could form the continuous ion pathway and guarantee the sufficient electrolyte penetration, shortening the ion diffusion length and accelerating the kinetics of electrochemical reactions. Therefore, the sodium‐ion storage performance including the rate capability and cycling stability can be significantly promoted. As a result, the fabricated 3D‐printed SICs based on N‐Ti_3_C_2_T*_x_* anode and AC cathode (Figure 8g) delivered a high areal/power density of 1.18 mWh cm^−2^/40.15 mW cm^−2^. All these findings revealed the advantage of 3D macroporous MXenes architecture on the energy storage systems with satisfying energy and power densities.

### 5.3 MXene‐Based Composites for Sodium‐Ion Storage

MXene‐based composite materials with excellent performance and well‐formed interfaces have been explored to resist the accumulation/agglomeration, optimize the charge transfer, and alleviate the volume expansion of the batteries. The performance of MXene‐based composites for SIBs in recent years is summarized and listed in **Table** **3**. In this review, on the basis of the preparation methods and mechanisms, MXene‐based composites are divided into three categories: growth of secondary materials on MXenes, self‐assembly for MXenes and other materials, and MXene‐based composites formed by in situ transformation reactions. First, due to the excellent electrical conductivity, highly hydrophilic surface and superior Young's modulus, MXenes have been considered as the ideal matrix for transition metal oxides, sulfides, selenides, phosphides, and alloy materials to improve their kinetics during electrochemical processes and cycling stability.^[^ ^95^ ^]^ Through the nucleation and growth, secondary materials can be in situ formed on the surface of MXenes. By contrast, in the self‐assembly method, prior to combination with MXenes, other materials are in their final form.^[^ ^6^, ^96^ ^]^ Finally, the MXene surface can be partially transformed into a secondary material through the in situ transformation reactions such as oxidation or sulfidation.^[^ ^97^ ^]^

**Table 3:** Comparison of MXene‐based composites for SIBs

Classification	Material	Long‐term capacity [mAh g^−1^]	Cycle number	Current density [A g^−1^]	Rate capability	Ref.
Growth on MXenes	Ti_3_C_2_T*_x_*/SnS	320	50	0.5	255 mAh g^−1^ at 1 A g^−1^	^[^ ^98^ ^]^
	Ti_3_C_2_T*_x_*/CoS	267	1700	2	272 mAh g^−1^ at 5 A g^−1^	^[^ ^100^ ^]^
	Ti_3_C_2_T*_x_*/CoNiO_2_	223	140	0.1	188.4 mAh g^−1^ at 3 A g^−1^	^[^ ^99^ ^]^
	Ti_3_C_2_T*_x_*/NaTi_2_(PO_4_)_3_	121	500	0.2	67 mAh g^−1^ at 2 A g^−1^	^[^ ^101^ ^]^
	Ti_3_C_2_/NiCoP	261.7	2000	1	240.1 mAh g^−1^ at 2 A g^−1^	^[^ ^102^ ^]^
	Ti_3_C_2_T*_x_*/Sb_2_O_3_	472	100	0.1	295 mAh g^−1^ at 2 A g^−1^	^[^ ^103^ ^]^
	Ti_3_C_2_T*_x_*/Sb	200	500	0.1	127 mAh g^−1^ at 2 A g^−1^	^[^ ^104^ ^]^
	Ti_3_C_2_T*_x_*/Bi_2_S_2_	155	250	0.5	168 mAh g^−1^ at 5 A g^−1^	^[^ ^105^ ^]^
	Ti_3_C_2_T*_x_*/MoS_2_	250.9	100	0.1	162.7 mAh g^−1^ at 1 A g^−1^	^[^ ^106^ ^]^
	Ti_3_C_2_T*_x_*/MoS_2_	331	70	0.1	488 mAh g^−1^ at 0.8 A g^−1^	^[^ ^107^ ^]^
	MoS_2_‐in‐Ti_3_C_2_	310	1600	1	241 mAh g^−1^ at 3 A g^−1^	^[^ ^108^ ^]^
	Ti_3_C_2_T*_x_*/MoSe_2_	434	200	1	250 mAh g^−1^ at 10 A g^−1^	^[^ ^109^ ^]^
	Ti_3_C_2_T*_x_*/VO_2_	280.9	200	0.1	206 mAh g^−1^ at 1.6 A g^−1^	^[^ ^110^ ^]^
Self‐assembly	Ti_3_C_2_/BP	100	200	0.1	67.3 mAh g^−1^ at 1 A g^−1^	^[^ ^113^ ^]^
	Ti_3_C_2_T*_x_*/FeS_2_	563	100	0.1	186 mAh g^−1^ at 10 A g^−1^	^[^ ^114^ ^]^
	Ti_3_C_2_T*_x_*/phosphorene	343	1000	1	193 mAh g^−1^ at 5 A g^−1^	^[^ ^115^ ^]^
	Ti_3_C_2_T*_x_*/SnS_2_	322	200	0.1	78 mAh g^−1^ at 2 A g^−1^	^[^ ^116^ ^]^
	Ti_3_C_2_T*_x_*/FePS_3_	676.1	90	0.1	449 mAh g^−1^ at 5 A g^−1^	^[^ ^117^ ^]^
	TiO_2_@Ti_3_C_2_T*_x_*	110	5000	0.96	68 mAh g^−1^ at 3.84 A g^−1^	^[^ ^118^ ^]^
	Ti_3_C_2_/PDDA‐BP	658	2000	1	461 mAh g^−1^ at 2 A g^−1^	^[^ ^119^ ^]^
	Ti_3_C_2_T*_x_*/CNT	345 mAh cm^−3^	500	0.1	89 mAh cm^−3^ at 5 A g^−1^	^[^ ^120^ ^]^
	Ti_3_C_2_T*_x_*/HC	272.3	1500	0.2	98.2 mAh g^−1^ at 2 A g^−1^	^[^ ^121^ ^]^
In situ transformation reaction	Ti_3_C_2_T*_x_*/TiO_2_	101	500	0.2	52 mAh g^−1^ at 2A g^−1^	^[^ ^126^ ^]^
	Ti_3_C_2_T*_x_*/TiO_2_	153	100	0.6	151.5 mAh g^−1^ at 1 A g^−1^	^[^ ^127^ ^]^
	Nb_2_CT*_x_*/Nb_2_O_5_	102	500	1	99 mAh g^−1^ at 2 A g^−1^	^[^ ^128^ ^]^
	MXene@NTP‐C	148	2000	1	102 mAh g^−1^ at 10 A g^−1^	^[^ ^129^ ^]^
	Ti_3_C_2_/Na_0.23_TiO_2_	56	4000	2	47 mAh g^−1^ at 3 A g^−1^	^[^ ^130^ ^]^
	Ti_3_C_2_/NTO	82	1900	2	78 mAh g^−1^ at 2 A g^−1^	^[^ ^131^ ^]^
	CoS_2_/CNT/TiO*_x_*N*_y_*	106	50	1	104 mAh g^−1^ at 2 A g^−1^	^[^ ^132^ ^]^

#### 5.3.1 Growth of Secondary Materials on MXenes

**Figure 9:**
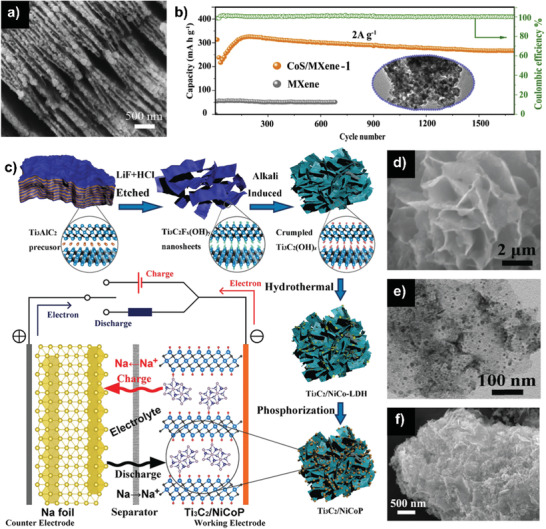
a) SEM images of Ti_3_C_2_T*_x_* flake. Reproduced with permission.^[^ ^98^ ^]^ Copyright 2017, Elsevier B.V. b) Cycling performances at 2 A g^−1^ (inset: TEM image of CoS/MXene composite after 1000 cycles). Reproduced with permission.^[^ ^100^ ^]^ Copyright 2018, Elsevier B.V. c) Schematic illustration of the synthesis process of the Ti_3_C_2_/NiCoP hybrid and schematic mechanism of half‐cells, d) SEM, and e) TEM images of Ti_3_C_2_/NiCoP hybrid. Reproduced with permission.^[^ ^102^ ^]^ Copyright 2019, Royal Society of Chemistry. f) field‐emission SEM image of MXene/Bi_2_S_3_. Reproduced with permission.^[^ ^105^ ^]^ Copyright 2020, Royal Society of Chemistry.

Fabrication of the composite consisting of MXenes and 0D nanoparticles is a feasible and effective way to develop advanced MXene‐based electrodes for SIBs. Through hydrothermal/solvothermal procedure, the composites of MXenes with SnS^[^ ^98^ ^]^ (**Figure** **9**a), CoNiO_2_,^[^ ^99^ ^]^ CoS,^[^ ^100^ ^]^ and NaTi_2_(PO_4_)_3_^[^ ^101^ ^]^ nanoparticles have been developed, where the ultrafine nanoparticles are homogenously embedded on MXene flakes. When applied in SIBs, the designed Ti_3_C_2_T*_x_*/CoS nanocomposites delivered a remarkable capacity of 267 mAh g^−1^ after 1700 cycles at 2 A g^−1^ (Figure 9b). The outstanding electrochemical performance can be ascribed to the synergistic effect of MXene substrate and CoS nanoparticles, in which MXenes provide the stable conductive network, prevent the agglomerate, and reduce the size of CoS nanoparticles, while CoS prevent the restack of MXene flakes. These desirable features provide more active sites for fast electrochemical reactions and shorten the distance of sodium‐ion diffusion, resulting in the superior rate performance and cycling stability. Remarkably, Zhao et al. synthesized a well‐designed Ti_3_C_2_/NiCoP interconnected structure via solvothermal method and subsequent in situ phosphorization reaction (Figure 9c), where NiCoP nanoparticles are homogenously embedded on the surface of 3D porous MXene networks (Figure 9d,e).^[^ ^102^ ^]^ The interconnected 3D MXene structure provided a 3D conductive channel for charge transfer processes and for electrolyte penetration, leading to the close contact between the electrolyte and electrode. The synergistic effect between MXene and NiCoP rendered a high structural stability, effectively prevented the aggregation and tolerated volume expansion. As a result, an impressive capacity of 261.7 mAh g^−1^ after 2000 cycles at 1 A g^−1^ for SIBs was obtained.

Besides these advances based on hydrothermal reaction, other techniques have also been tried to obtain the MXene/0D heterostructures. For example, Guo et al. prepared Ti_3_C_2_T*_x_*/Sb_2_O_3_ composites via hydrolysis route with Sb_2_O_3_ nanoparticles (50 nm) uniformly distributed in the MXene networks.^[^ ^103^ ^]^ MXenes provide the highly ionic and electronic conductive network for Sb_2_O_3_ nanoparticles and Sb_2_O_3_ nanoparticles prevent the MXenes flakes from restacking and serve as sodium‐ions reservoir. Meanwhile, the voids generated during preparation process could accommodate the volume expansion. As expected, the hybrid anodes delivered a capacity of 472 mAh g^−1^ after 100 cycles at 0.1 A g^−1^ and 295 mAh g^−1^ at 2 A g^−1^. Chen et al. decorated Ti_3_C_2_T*_x_* MXenes with Sb nanoparticles (5–10 nm) through a solution‐phase method.^[^ ^104^ ^]^ The hybrid materials delivered a capacity of 200 mAh g^−1^ after 500 cycles at 0.1 A g^−1^. Yang et al. achieved Ti_3_C_2_T*_x_*/Bi_2_S_2_ composites with a sandwich‐like stereochemical structure (Figure 9f), alleviating the volume expansion and increasing active areas.^[^ ^105^ ^]^

**Figure 10:**
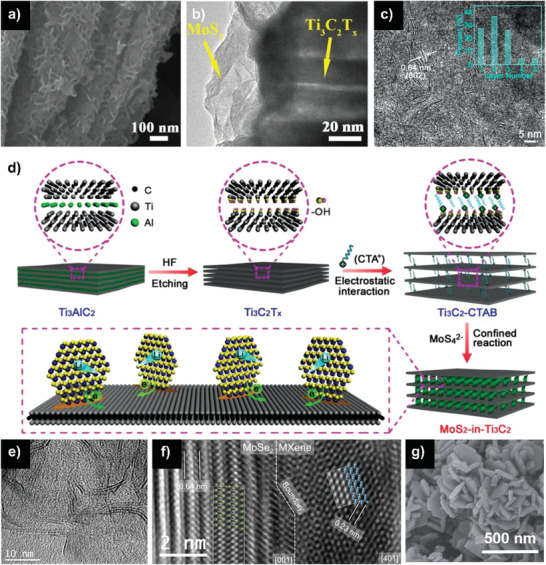
a,b) SEM and TEM images of Ti_3_C_2_T*_x_*/MoS_2_ composite. Reproduced with permission.^[^ ^107^ ^]^ Copyright 2019, Royal Society of Chemistry. c) HRTEM images of the MoS_2_‐in‐Ti_3_C_2_ hybrids (inset showing the corresponding SAED pattern and the layer number distribution of MoS_2_ nanocrystals) and d) schematic illustration for the fabrication of the MoS_2_‐in‐Ti_3_C_2_ hybrids. Reproduced with permission.^[^ ^108^ ^]^ Copyright 2018, WILEY‐VCH GmbH. e,f) TEM images of MXene/MoSe_2_ heterojunction. Reproduced with permission.^[^ ^109^ ^]^ Copyright 2019, Elsevier B.V. g) SEM images of the MXene/VO_2_. Reproduced with permission.^[^ ^110^ ^]^ Copyright 2019, Royal Society of Chemistry.

Coupling MXenes with other 2D materials, especially with MoS_2_, have been reported in SIBs.^[^ ^106^ ^]^ With a quantity of MoS_2_ nanosheets deposited in MXene layers (**Figure** **10**a,b), Ti_3_C_2_T*_x_*/MoS_2_ heterostructures exhibited a capacity of 331 mAh g^−1^ after 70 cycles at 0.1 A g^−1^ for SIBs.^[^ ^107^ ^]^ Impressively, Ma et al. reported the confined synthesis of one to three‐layered MoS_2_ nanocrystals in Ti_3_C_2_ interlayer (Figure 10c) by CTAB‐directed growth assisted with thermal treatment (Figure 10d).^[^ ^108^ ^]^ Through the unique 2D nanospace confinement effect and Mo—C covalent bond, the MoS_2_ nanocrystals were tightly anchored in the interlayer, significantly strengthening the structural stability of composites. Furthermore, the as‐obtained MoS_2_‐in‐Ti_3_C_2_ hybrids with high power MXene and high energy MoS_2_ created strong coupling as well as substantial edges and active sites. As a consequence, the fabricated SIBs delivered an excellent cycling performance with a high capacity of 310 mAh g^−1^ at 1 A g^−1^ maintaining 1600 times and ultrahigh rate performance of 241 mAh g^−1^ at 3 A g^−1^. Because it is very hard for traditional diffusion‐controlled electrochemical behavior to achieve such high rate performance and the pseudocapacitance‐dominated contribution is usually much faster and more stable, this ultrahigh rate capability may be ascribed to the pseudocapacitance‐dominated storage mechanism. Later, the disappearance of the peak at 1.0 V in CV curves confirmed this hypothesis. Specifically, at a scan rate of 2.0 mV s^−1^, the capacitive‐dominated contribution reached as high as 84.7%. Besides, few‐layer 2D MoSe_2_ nanosheets were also successfully grown on the surface of Ti_3_C_2_T*_x_* (Figure 10e).^[^ ^109^ ^]^ Due to the van der Waals interaction, the MoSe_2_ and MXenes are combined closely near the boundary (Figure 10f), effectively restraining the volume change during the sodium‐ions insertion/extraction courses. As anodes for SIBs, the materials attained a capacity of 434 mAh g^−1^ after 200 cycles at 1 A g^−1^. In addition, Wu et al. reported a flower‐like structure of Ti_3_C_2_T*_x_*/VO_2_ hybrid (Figure 10g) with high specific surface area, permitting the high degree of electrolyte penetration and shortening the diffusion pathway of sodium ions.^[^ ^110^ ^]^ It should be noted that the VO_2_ flakes effectively prevented the restacking of MXene nanosheets. The enhanced electrical conductivity due to the conductive MXene matrix further improved the sodium‐ion diffusion kinetics. Finally, this hybrid showed a high reversible capacity of 280.9 mAh g^−1^ at 0.1 A g^−1^ over 200 cycles when used as an anode in SIBs. Ex situ XRD and TEM assessments validated the amorphous transformation of VO_2_ during the process of sodium‐ion intercalation, reducing the extent of volume expansion and further improving the rate performance.

Except for the layer re‐stacking issue for MXenes, air‐oxidation is another bottleneck problem that severely influences their energy storage performance. Considering this, Zhang et al. designed an effective way to obtain the carbon‐coated MXenes through the self‐polymerization of dopamine all over the Ti_3_C_2_T*_x_* sheets.^[^ ^111^ ^]^ The polymeric layer not only accelerates the transformation of nanosheets into 3D architecture, but also forms a uniform thin carbon coating layer after the carbonization treatment, simultaneously inhibiting the layer re‐stacking and air‐oxidation. In the application of SIBs, the long cycle capacity of 91.7% after 3000 cycles at 1 A g^−1^ is obtained. Galvanostatic intermittent titration technique and EIS were considered to understand the underlying mechanism of sodium‐ion storage, revealing the high diffusion coefficient and low electron transfer resistance of electrode. The strategy described here could also be expected to prepare MXene‐based composites for other perspective energy applications.

#### 5.3.2 Self‐Assembly for MXenes and Other Materials

**Figure 11:**
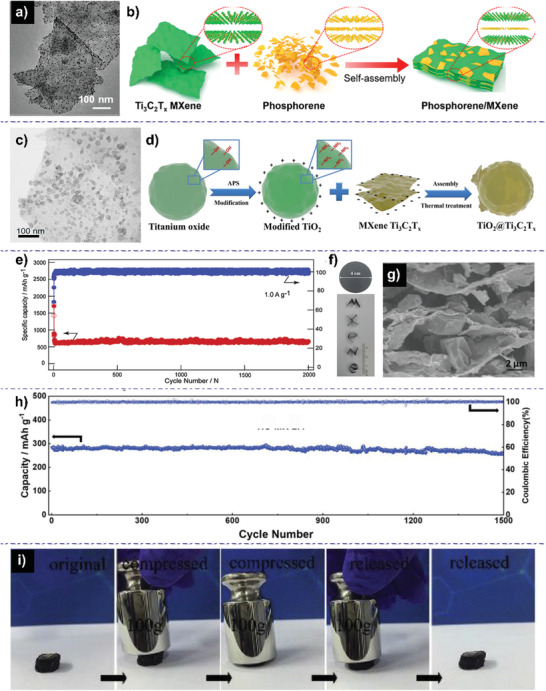
a) TEM image of the MXene/BPQD composite. Reproduced with permission.^[^ ^112^ ^]^ Copyright 2018, WILEY‐VCH GmbH. b) Schematic illustration of the synthetic processes for MXene/phosphorene hybrid structure. Reproduced with permission.^[^ ^115^ ^]^ Copyright 2020, American Chemical Society. c) TEM image of MXene/SnS_2_. Reproduced with permission.^[^ ^116^ ^]^ Copyright 2017, Elsevier B.V. d) Schematic illustration of synthetic process for TiO_2_@Ti_3_C_2_T*_x_* material. Reproduced with permission.^[^ ^118^ ^]^ Copyright 2018, Published by Elsevier B.V. e) Long cycling performance at 1.0 A g^−1^ for 2000 cycles for Ti_3_C_2_/PDDA‐BP heterostructures electrode. Reproduced with permission.^[^ ^119^ ^]^ Copyright 2019, Elsevier Ltd. f) Digital photo and g) SEM images of the flexible MXene/HC film, f) the shaped letters of the word “MXene”, and h) cycle performance of all electrodes at 0.2 A g^−1^ for MXene/HC. Reproduced with permission.^[^ ^121^ ^]^ Copyright 2019, WILEY‐VCH GmbH. i) Photographs of a typical compression process of freestanding‐GpTiC. Reproduced with permission.^[^ ^122^ ^]^ Copyright 2019, Published by Elsevier Ltd.

Usually, due to the unique desorption behavior of MXenes, researchers have successfully obtained the composites by mixing the two aqueous solutions coupled with longtime stirring and/or sonication. After the self‐assemble process, the composites were collected by centrifugation or vacuum‐assisted filtration. For instance, Meng et al. fabricated the Ti_3_C_2_/black phosphorus quantum dots (BPQDs) nanocomposites with BPQDs (sub‐10 nm) homogenously anchored on Ti_3_C_2_ nanosheets (**Figure** **11**a).^[^ ^112^ ^]^ It was found that strong covalent interaction due to P—O—Ti bonds is formed at the interfaces between them, inducing the atomic charge polarization and improving the pseudocapacitive charge storage. Especially, the composite electrode exhibited the battery‐capacitive dual‐model energy storage mechanism (DMES) with fast, complete, and stable alloying reaction as a typical battery‐type anode and increased pseudocapacitive capacity because of the pseudocapacitive component of MXenes. Moreover, the reinforced battery‐capacitive DMES guaranteed the outstanding comprehensive performance of batteries. Li et al. also prepared Ti_3_C_2_/BP composites with Ti_3_C_2_ nanoflakes and black phosphorus (BP) nanoparticles.^[^ ^113^ ^]^ As expected, when used as anodes for SIBs, the composites delivered a capacity of 100 mAh g^−1^ after 200 cycles at 0.1 A g^−1^. Du et al. reported the composites with FeS_2_ nanodots (10 nm) uniformly covered on the surface of MXenes.^[^ ^114^ ^]^ For sodium‐ion storage, the hybrid presented a reversible capacity of 563 mAh g^−1^ after 100 cycles at 0.1 A g^−1^. The electrochemical kinetics characterizations including EIS and CV were performed to analyze the charge‐storage mechanism, indicating that the hybrid have the smaller charge transfer resistance and enhanced capacitive character. These electrochemical behaviors were attributed to the large surface area and extraordinary electrical conductivity of the unique Ti_3_C_2_T*_x_*/FeS_2_ heterostructure.

Meanwhile, hybridization of MXenes with other 2D materials was also synthesized by this liquid–solid‐phase self‐assembly. A Ti_3_C_2_T*_x_*/phosphorene hybrid anode for fast and stable sodium storage (Figure 11b) has been reported.^[^ ^115^ ^]^ The composites not only accommodate the volume expansion but also increase the migration of sodium ions and electrons. Moreover, MXenes with F‐terminated groups give rise to the formation of F‐rich solid electrolyte interphase on the anode surface. DFT calculations also demonstrated that the heterostructures, especially Ti_3_C_2_F_2_/phosphorene, have enhanced sodium affinities and diffusion kinetics. Consequently, the hybrid electrode attained a superior capacity (343 mAh g^−1^ at 1 A g^−1^ with a retention of 87% over 1000 cycles). Wu et al. constructed a heterolayered structure of Ti_3_C_2_T*_x_* flakes composited with SnS_2_ nanoplates (Figure 11c).^[^ ^116^ ^]^ The addition of SnS_2_ nanoplates offers substantial diffusion paths and sodium‐ion absorption active sites, leading to the fast electrochemical kinetics. When applied as anodes for SIBs, a reversible capacity of 120 mAh g^−1^ after 125 cycles at 1 A g^−1^ was achieved at an extreme temperature of 0 °C. Ding et al. fabricated Ti_3_C_2_T*_x_*/FePS_3_ hybrids with few‐layered FePS_3_ nanosheets homogenously coated by MXenes, providing a capacity of 676.1 mAh g^−1^ at 0.1 A g^−1^ and 527.7 mAh g^−1^ at 0.5 A g^−1^ after 90 cycles for SIBs, respectively.^[^ ^117^ ^]^ The high charge capacity is benefited from the unique 2D/2D heterojunction structure, promoting the capacitance kinetics in the high‐rate charge–discharge processes and buffering the volume expansion.

On the other hand, as we all know, MXenes are negatively charged due to the existence of surface groups. Therefore, various MXene‐based composites have been reported with excellent sodium storage properties by self‐assembly based on electrostatic attraction. For example, Guo et al. fabricated TiO_2_@Ti_3_C_2_T*_x_* composites by self‐assembly negatively charged MXene nanosheets and positively charged TiO_2_ spheres as anodes for SIBs.^[^ ^118^ ^]^ As shown in Figure 11d, at first, the as‐prepared TiO_2_ spheres were NH_2_‐functionalized by 3‐aminopropyltrimethoxysilane to endow the surface of TiO_2_ positively charged and then attracted to the surface of MXene nanosheets until the charge was balanced. Herein, MXene shells protected the TiO_2_ spheres from pulverization during the charge–discharge processes and contributed to the formation of the ultra‐stable solid‐electrolyte interface films. Owing to the robust structure and boosted pseudocapacitance, the battery obtained a capacity of 110 mAh g^−1^ after 5000 cycles at 0.96 A g^−1^ without obvious capacity fading. Zhao et al. synthesized Ti_3_C_2_/PDDA‐BP heterostructures based on poly(diallyl dimethyl ammoniumchloride) (PDDA)‐modified layered black phosphorene (BP).^[^ ^119^ ^]^ The modification of PDDA not only made BP positively charged but also improved their dispersity and stability. As expected, the heterostructure electrodes for SIBs exhibited an ultralong cycling stability of 658 mAh g^−1^ within 2000 cycles at 1 A g^−1^ with 0.05% capacity decay of per cycle (Figure 11e). More importantly, the Ti_3_C_2_/PDDA‐BP heterostructures always show the larger capacitive contribution than the mechanically mixed Ti_3_C_2_/BP, which is mainly ascribed to strong interactions between Ti_3_C_2_ and PDDA‐BP. This interaction was certified by DFT calculations, effectively decreasing the binding energy and facilitating sodium storage kinetics.

Furthermore, considering their 2D layer structure and easy construction of film electrode, MXene‐based materials have attracted extensive research interest as freestanding, flexible electrodes for next‐generation sodium storage. Xie et al. first reported the freestanding, porous Ti_3_C_2_T*_x_*/carbon nanotube (CNT) films for sodium storage by self‐assembly of negatively charged Ti_3_C_2_T*_x_* and positively charged CNTs with CTAB modification, which achieved a capacity of 89 mAh cm^−3^ at high current density of 5 A g^−1^.^[^ ^120^ ^]^ Particularly, Sun et al. reported the freestanding and flexible hard carbon (HC) films (Figure 11f) for SIBs bonded by Ti_3_C_2_T*_x_* MXenes, where HC particles are embedded in the 3D conductive network constructed by MXene flakes (Figure 11g), effectively stabilizing the structure of electrode and accommodating the volume expansion of HC during electrochemical processes.^[^ ^121^ ^]^ Because of the elimination of inactive electrochemically components, the MXene‐bonded electrodes showed enhanced capacities than the conventional PVDF‐bonded HC electrodes. After 1500 cycles at 0.2 A g^−1^, a reversible capacity of 272.3 mAh g^−1^ can still be obtained with no capacity loss (Figure 11h). Zhang et al. obtained an elastic freestanding structure for SIBs by assembling Ti_3_C_2_T*_x_* sheets with rGO and cellulose nanofibers (CUNFs) (GpTiC).^[^ ^122^ ^]^ As depicted in Figure 11i, when deformed to a large extent, they can be recovered to original state. At a current density of 1 A g^−1^, the resulting electrodes delivered a high capacity retention of 84.8% and 75.8% after 1000 and 2000 cycles, respectively.

#### 5.3.3 MXene‐Based Composites Formed by In Situ Transformation Reactions

**Figure 12:**
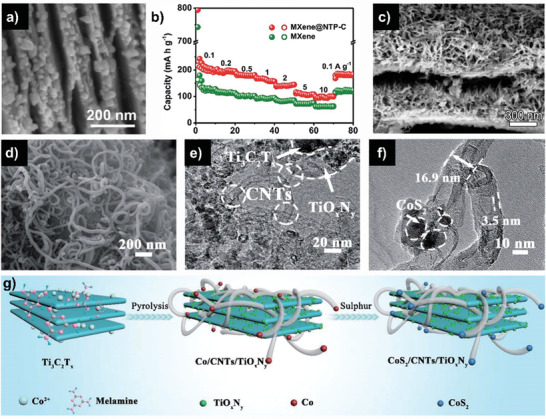
a) SEM image of Ti_3_C_2_/TiO_2_. Reproduced with permission.^[^ ^126^ ^]^ Copyright 2018, Elsevier Ltd. b) Rate capability of MXene@NTP‐C and MXene at varied current densities of 0.1–10 A g^−1^. Reproduced with permission.^[^ ^129^ ^]^ Copyright 2018, Royal Society of Chemistry. c) SEM image of Ti_3_C_2_/Na_0.23_TiO_2_ composite. Reproduced with permission.^[^ ^130^ ^]^ Copyright 2018, Elsevier Ltd. d) SEM, e,f) TEM images, and g) schematic of the synthetic process of CoS_2_/CNTs/TiO*_x_*N*_y_*. Reproduced with permission.^[^ ^132^ ^]^ Copyright 2020, Royal Society of Chemistry.

Considering that pristine MXene phase can be completely transformed into other materials such as NaTi_1.5_O_8.3_,^[^ ^123^ ^]^ Na_2_Ti_3_O_7_@C,^[^ ^124^ ^]^ or TiO_2_/C^[^ ^125^ ^]^ through oxidation or alkalization processes, with careful design and reaction, great advances in the transformation strategies have been made to in situ obtain the MXene‐based composites. For example, Yang et al. constructed accordion‐like Ti_3_C_2_/TiO_2_ nanohybrid with expanded interspacing by facile hydration strategy, where TiO_2_ nanoparticles are in situ formed and homogenously decorated on the surface of resultant MXenes (**Figure** **12**a).^[^ ^126^ ^]^ First, the interlayer spacing of MXene has been enlarged, proving more sodium‐ion adsorption active sites and reducing the barriers of sodium‐ions mobility. Second, the electrical conductivity was improved, rendering the pathways for fast electron transfer. Third, the in situ‐formed TiO_2_ nanoparticles provided extra capacity for highly efficient sodium‐ion storage. Benefiting from these structural and componential advantages, the nanohybrid delivered a capacity of 101 mAh g^−1^ after 500 discharge–charge cycles at 0.2 A g^−1^ in SIBs application. Wang et al. also obtained Ti_3_C_2_/TiO_2_ composites with expanded interlayer spacing by calcination process of the preintercalated pristine MXene with TMAOH under the atmosphere of N_2_.^[^ ^127^ ^]^ The weight ratio of MXenes, TiO_2_, and amorphous carbon were calculated as 38.5, 55.9, and 5.6 wt%, respectively, suggesting that about 50% MXenes have been oxidized. The surface reaction of TiO_2_ nanoparticles could contribute to the pseudocapacitance contribution. When evaluated as anodes for SIB, the composites exhibited a capacity of 153 mAh g^−1^ after 100 cycles at 0.6 A g^−1^. Du et al. synthesized Nb_2_CT*_x_*/Nb_2_O_5_ composites by mild hydrothermal approach, in which MXenes are partially oxidized to Nb_2_O_5_ in the process.^[^ ^128^ ^]^ Thanks to the hybrid pseudocapacitance including the surface‐controlled pseudocapacitance and intercalation pseudocapacitance at the high and low potential, respectively, the composite rendered a capacity of 102 mAh g^−1^ at 1 A g^−1^ after 500 cycles for SIBs.

Other than the partial oxidization of MXenes to achieve the corresponding MXene/metal oxide composites, MXenes can be acted as the titanium source for the fabrication of a new material and simultaneously in situ assembled with MXenes. Yang et al. reported an anode material denoted as MXene@NTP‐C for SIBs, where NaTi_2_(PO_4_)_3_ cubes are uniformly covered on Ti_3_C_2_ nanosheets and coated with a carbon layer through in situ solvothermal transformation followed by calcination.^[^ ^129^ ^]^ Due to the nanoscale integration of MXene and NaTi_2_(PO_4_)_3_, the cells showed a superior rate capacity of 102–208 mAh g^−1^ at 10–0.5 A g^−1^ (Figure 12b). Electrochemical and kinetic measurements suggested that the excellent performance can be owing to the dual‐mode accommodation of sodium into the battery‐type NaTi_2_(PO_4_)_3_ and pseudocapacitance‐type MXene. Huang et al. proposed the sandwich‐like Ti_3_C_2_/Na_0.23_TiO_2_ architecture consisting of short nanobelts on nanosheets (Figure 12c) by one‐step in situ transformation of Ti_3_C_2_ in the NaOH solution, demonstrating a remarkable sodium storage performance of a 56 mAh g^−1^ after 4000 cycles at 2 A g^−1^.^[^ ^130^ ^]^ In fact, this sandwich‐like composites were composed of 1D ultrathin nanobelts, 2D conductive nanosheets, and 3D sandwich‐like structure, effectively relieving the strain of electrodes and facilitating the charge transport as well as protecting the agglomeration of electrode materials. In addition, Sun et al. explored a two‐step hydrothermal method for preparation of hybrid NaTi_8_O_13_/NaTiO_2_ nanoribbons in situ formed on Ti_3_C_2_ (designed as Ti_3_C_2_/NTO) surface.^[^ ^131^ ^]^ The well‐preserved 2D architecture could guarantee the high conductivity and large electrode/electrolyte contacting area as well as accelerate the sodium‐ion migration. Besides, the robust whole structure can accommodate the volume expansion during repeating charge/discharge processes and ease the peeling off of NTO. Consequently, a capacity of 82 mAh g^−1^ after 1900 cycles at 2 A g^−1^ was obtained for SIBs.

Recently, Tao et al. obtained the material of CoS_2_/CNT/TiO*_x_*N*_y_* where a composite of CoS_2_, TiO*_x_*N*_y_* nanoparticles, and CNT are uniformly decorated on the surface of MXene (Figure 12d–f).^[^ ^132^ ^]^ In the two‐step synthetic processes (Figure 12g), cobalt ions were reduced to cobalt nanoparticles and as catalysis to in situ form CNTs on the MXenes. Meanwhile, TiO*_x_*N*_y_* nanoparticles were also synthesized and covered on the MXenes. When the composites used as anodes for SIBs, they showed a capacity of 106 mAh g^−1^ after 50 cycles at 1 A g^−1^. The unique reaction mechanism of composite was explored through in situ Raman and ex situ XPS analysis, indicating that the active sulfates could serve as the intermediates during the reaction processes. Although the performance is not satisfied, the discovery of transformation of MXenes into CNTs under the catalysis of metal is very meaningful for advanced materials in future.

## 6 Conclusion and Perspectives

Electrochemical sodium storage technologies have attracted increasing attention due to the relatively low cost and virtually infinite resources of sodium. Numerous significant achievements have been acquired on account of the prompt development of relevant engineering and materials. As one of the hottest materials in recent years, MXenes have been intensively investigated in the storage field due to their extraordinary physical and electrochemical properties.

In this review, we have highlighted the recent progress on the synthesis, structures, electronic properties, and intercalation chemistries of MXene‐based materials and their applications in SIBs, SSBs, and SICs based on the theories and experiments. On the one hand, the synthetic strategies for pure MXenes such as F‐containing and F‐free etching method could result in different surface groups and interlayer spacing, strongly influencing their structures and properties. The association between them is systematically introduced. Besides, the application of pure MXenes including the multilayer MXenes, single‐/few‐layer MXenes, MXenes with expanded interlayer spacing, and 3D porous structures in the sodium‐ion storage is comprehensively summarized. On the other hand, to make the most of MXenes, a variety of nanomaterials from 0D to 2D have been successfully combined with MXenes, forming the MXene‐based composites with unique architectures. Based on the synthetic strategies, MXene‐based composites are classified into three types: growth on MXenes, self‐assembly, and in situ transformation reaction. The enhanced sodium‐ion storage performance can be ascribed to the synergistic effects between MXenes and the other materials. First, MXenes form the robust and conductive transfer pathway for electrons/ions, dramatically facilitating the transportation kinetics during the electrochemical reactions. Second, the high specific surface area of MXenes matrix ensures the uniform distribution of other active materials, effectively accommodating the volume expansion. Third, the hybrid materials can provide more active sites for sodium‐ion absorption and prevent aggregation of individual nanomaterials. Fourth, the established 3D porous architecture effectively shortens the diffusion path of sodium ions and accelerates the penetration of electrolytes. Therefore, the fabricated sodium‐ion storage systems could deliver the higher rate capability and better cycling stability than the reference devices. Although MXene‐based materials for electrochemical sodium‐ion storage have been extensively studied and show expected prospects, there are still several critical obstacles that need to be further optimized for their practical applications as shown below:

1) Generally, the dominated acid etching methods obtaining MXenes have the low yield and high risk. In this regard, it is important to develop a controllable, efficient, green, and safe synthetic strategy to synthesize MXenes with controlled number of layers, tunable surface groups, enlarged interlayer spacing, and outstanding quality. F‐free and bottom‐up strategies like chemical vapor deposition, atomic layer deposition should be given more attention in the future.
2) As mentioned earlier, the surface chemistries of MXenes could dramatically influence the properties of MXene‐based materials. Different etchant could result in MXenes with different surface groups. So far, MXenes without any surface groups have not been obtained. Besides, the precise surface chemistries of MXenes and the interaction between the surface groups and materials are still not very clear. It has been suggested that the bare/‐O terminated MXenes have better physicochemical properties. Thus, more researches are necessary to be carried out to regulate the surface chemistries and explore their application in the electrochemical storage.
3) Little works have been published on the MXenes beyond Ti_3_C_2_T*_x_*. Looking for new MXenes with different functions to develop sodium‐ion storage has great potential. For instance, according to the calculations, TiC_3_ exhibits better anode material performance than Ti_3_C_2_ due to the large absorption area and strong adsorption ability for sodium ions.^[^ ^47^ ^]^ Ti_2_C MXenes give a capacity of 536.84 mAh g^−1^.^[^ ^50^ ^]^ The heterostructures of VS_2_ with Ti_2_CO_2_ and V_2_CO_2_ have huge capacities and small OCVs simultaneously.^[^ ^18^ ^]^ We also hope that these materials can be applied in the promising directions through further optimization.
4) The long‐term development of MXene‐based materials is strongly hindered by the restacking problem and instability of MXenes in the oxygen environments. In order to alleviate this situation, efforts can be focused on the rational structural design and accurate morphological control of the electrodes such as the 3D porous architectures, aerogels, and coating techniques.
5) Theoretical studies must take into consideration of the nonuniform, incomplete, and mixed coverage of surface groups and stacked multilayer of MXenes to precisely predict their properties and in turn guide experiments. Considering these superior complexities, it is necessary to combine the simulations with the advanced computations including machine learning, classical molecular dynamics, and high‐throughput computation to sufficiently understand the surface chemistries and interfaces. Moreover, comprehensive theories and predictive models as well as the computing software and hardware should be further developed to narrow the gap between the predictions and experimental results at low cost.
6) The sodium‐ion storage mechanisms underlying the electrochemical reaction and the component/structural evolution during charge/discharge processes should be in‐depth investigated through in situ works, such as in situ XRD, in situ Raman, in situ scanning electron microscopy (SEM), in situ TEM, and so on. In addition, further optimizing current sodium‐ion storage systems including the electrode configuration, electrolyte type, electrode/electrolyte interactions, and interfaces is also important for desirable performance.

The engineering of nanostructured electrode materials is promising in the applications, which could lead to the enhanced electrochemical performance due to their unique nanostructures, large surface areas, and tunable compositions. Ether‐based electrolytes could provide a higher initial CE and longer cycling life in SIBs, while the aqueous electrolyte is a good choice for green, safe, and scale sodium‐ion storage systems. Besides, a more stable and thinner electrode/electrolyte interface is highly expected to buffer the volume change and improve the sodium storage performance. To satisfy the commercial requirements of SIBs in large‐scale energy storage system, comparable performance to state‐of‐the‐art LIBs such as long cycle life and high energy density as well as the lower production cost should be intensively progressed. We believe that success in addressing above‐mentioned problems will contribute to the future evolution of MXene‐based materials in electrochemical sodium‐ion storage applications.

## Conflict of Interest

The authors declare no conflict of interest.
